# Minimally invasive radical prostatectomy versus open radical prostatectomy: A systematic review and meta-analysis of randomized control trials

**DOI:** 10.1016/j.clinsp.2025.100636

**Published:** 2025-04-27

**Authors:** Caio Felipe Araujo Matalani, Mateus Silva Santos Costa, Marcelo Ribeiro da Rocha, Roberto Iglesias Lopes, Thalita Bento Talizin, José Bessa Júnior, William Carlos Nahas, Leopoldo Alves Ribeiro-Filho, Caio Vinicius Suartz

**Affiliations:** a Division of Urology, Institute of Cancer of São Paulo, Universidade de São Paulo (USP), São Paulo, SP, Brazil; b Department of Health, Universidade Estadual de Feira de Santana (UEFS), Feira de Santana, BA, Brazil; c CHU de Québec-Université Laval, Quebec City, Quebec, Canada; d Urology Department, Northern Ontario School of Medicine, Thunder Bay, Ontario, Canada

**Keywords:** Prostate cancer, Robot-assisted radical prostatectomy, Open radical prostatectomy, Randomized Control Trial, Meta-Analysis

## Abstract

**Objective:**

To evaluate the comparative outcomes of Minimally Invasive Radical Prostatectomy (MIRP) versus Open Radical Prostatectomy (ORP) to treat localized prostate cancer, using only Randomized Controlled Trials (RCTs) to ensure high-quality evidence.

**Method:**

A systematic review and meta-analysis were conducted in accordance with PRISMA guidelines, focusing solely on RCTs comparing MIRP (robot-assisted or laparoscopic surgery) and ORP. Literature searches across multiple databases, including Cochrane Library, Medline, Embase, Lilacs, Scopus, Web of Science, NIH, Clinical Trials, and EU Clinical Trials Register, identified studies meeting predefined PICOT criteria. Four RCTs met inclusion criteria ‒ two representing the same cohort of patients ‒ and were analyzed for perioperative, functional, and oncologic outcomes. Quality assessment utilized the ROB-2 tool to gauge the risk of bias.

**Results:**

Three RCTs encompassing 1051 patients were analyzed. MIRP demonstrated statistically significant benefits over ORP in terms of reduced perioperative blood loss (Standardized Mean Difference [SMD = -3.058], *p* = 0.006), lower transfusion rates (Odds Ratio [OR = 0.137]; *p* = 0.009), and fewer overall complications (OR = 0.465; *p* = 0001). However, no significant differences were found in long-term oncologic and functional outcomes, including urinary continence and erectile function. Positive surgical margins and additional treatments also did not differ significantly between groups.

**Conclusion:**

This systematic review and meta-analysis of RCTs indicated that MIRP offers perioperative advantages over ORP, supporting its role as a safe and effective option for localized prostate cancer.

## Highlights

• Minimally invasive surgery reduces blood loss and transfusions vs. open surgery.
• Fewer complications occur with minimally invasive than with open prostate surgery.
• Urinary continence and erectile function are similar in both surgical approaches.
• Cancer control outcomes are comparable between minimally invasive and open surgery.
• More studies are needed to confirm the long-term benefits of minimally invasive surgery.

## Introduction

Prostate cancer is an important global health issue, being the second most common cancer in males worldwide.^1^ In 2022, it was estimated that there were 268,490 new cases of this cancer and 34,500 deaths in the United States.^2^ As a result of the widespread use of PSA testing, there has been an increase in the incidence of localized disease, making the discussion about early stages even more significant.

For localized prostate cancer, primary treatment options include radiotherapy and surgery.^3^ The decision between these treatments is influenced by manifold factors such as the patient's age, comorbid conditions, personal preferences, and the expertise and resources available at the treating center. Both radiotherapy and radical prostatectomy have shown effectiveness in managing localized disease, with comparable long-term survival outcomes.^4^

The surgical treatment of prostate cancer has undergone significant advancements in recent decades. Open Radical Prostatectomy (ORP) remains the standard approach, but the advent of minimally invasive techniques, such as Laparoscopic Prostatectomy (LP), and, since Binder et al. (2001), Robot-Assisted Radical Prostatectomy (RARP), has revolutionized surgical management, being the primary surgical approach in many countries.^5^^,^^6^ These advancements have improved functional outcomes, reduced perioperative complications, decreased blood loss and transfusion rates, and shorter patient hospital stays.^7^^,^^8^

Despite treatment progress, few Randomized Controlled Trials (RCTs) compare Minimally Invasive Radical Prostatectomy (MIRP) with ORP for localized prostate cancer. A significant RCT conducted by Nahas et al. (2024) recently furnished valuable new data, which could play a pivotal role in informing clinical recommendations and healthcare policies.^9^

Therefore, the authorsconducted a systematic review that includes only RCTs to enhance the accuracy of meta-analysis and provide a stronger evidence base for clinical decision-making and the development of therapeutic guidelines.

## Material and methods

### Literature search

The research was completed in full compliance with the Preferred Reporting Items for Systematic Reviews and Meta-Analysis (PRISMA)^10^ statement on August 24, 2024. It was registered in the PROSPERO international database as a prospectively registered systematic review (CRD 42,024,568,284).

A research question was formulated based on the Patient-Index test-Comparator-Outcome-Study (PICOS) criteria, as follows^11^: Which type of surgical approach yields better oncological and functional outcome: robotic-assisted or open approach?

The search strategy was: (("Prostatectomies" OR "Prostatectomy, Suprapubic" OR "Prostatectomies, Suprapubic" OR "Suprapubic Prostatectomy" OR "Prostatectomy, Retropubic" OR "Prostatectomies, Retropubic" OR "Retropubic Prostatectomies" OR "Retropubic Prostatectomy") AND ("Procedure, Robotic Surgical" OR "Robotic Surgical Procedure" OR "Surgical Procedure, Robotic" OR "Robot Surgery" OR "Robot Surgeries" OR "Surgery, Robot" OR "Robot-Assisted Surgery" OR "Robot Assisted Surgery" OR "Robot-Assisted Surgeries" OR "Surgery, Robot-Assisted" OR "Robot-Enhanced Procedures" OR "Procedure, Robot-Enhanced" OR "Robot Enhanced Procedures" OR "Robot-Enhanced Procedure" OR "Surgical Procedures, Robotic" OR "Robotic-Assisted Surgery" OR "Robotic Assisted Surgery" OR "Robotic-Assisted Surgeries" OR "Surgery, Robotic-Assisted" OR "Robot-Enhanced Surgery" OR "Robot Enhanced Surgery" OR "Robot-Enhanced Surgeries" OR "Surgery, Robot-Enhanced" OR "RARP")) AND “Laparoscopic Prostatectomy”.

The authorssearched the following databases up to July 2024:

1. Cochrane Central Register of Controlled Trials (CENTRAL) 2020, Issue 3, in the Cochrane Library;
2. MEDLINE via Ovid (from 1946);
3. Embase via Ovid (from 1974);
4. LILACS (Latin American and Caribbean Health Science Information database, from 1982);
5. Scopus, Elsevier's citation tool (from 2004);
6. 6 Web of Science/Web of Knowledge (Clarivate and Thomson Reuters) (from 1900); and the following trial registries:
  1. National Institutes of Health (NIH - Pubmed) (https://pubmed.ncbi.nlm.nih.gov/);
  2. ClinicalTrials.gov (http://www.clinicaltrials.gov);
  3. Brazilian Registry of Clinical Trials (ReBEC) (https://ensaiosclinicos.gov.br/);
  4. EU Clinical Trials Register (http://www.clinicaltrialsregister.eu).

The authorsalso checked the bibliographies of the included studies for additional references to relevant trials.

The authorsincluded just randomized clinical trials. Meeting abstracts, reviews, case reports, letters to the editor, retrospective cohorts, and editorials were excluded.

### Study screening and selection

Six independent authors reviewed all retrieved records. Discrepancies were resolved through discussion. The full text of the reviewed papers was selected if deemed relevant to the present review.

### PICOT framework

The authorsincluded studies that met the following criteria: 1) Patients (P) adults over 18-years old with clinically localized prostate cancer; 2) The intervention (I) tested was the Robot-Assisted Radical Prostatectomy (RARP) or Laparoscopic Radical Prostatectomy (LRP), and the Comparator (C) was the open Retropubic Radical Prostatectomy (ORP). The primary Outcome (O) evaluated was functional and oncological results, and the search was done without time restrictions, selecting only randomized controlled Trials (T). Authors of the studies were contacted as necessary to request information regarding 2 × 2 tables or other relevant data needed for meta-analytical purposes.

### Data extraction and endpoints

All the variables were inputted into an Excel spreadsheet for analysis and cross-checked by another author. The mean and standard deviation for continuous variables were recorded from the included studies. For variables reported as median and interquartile range, the original data were converted to mean and standard deviation.^12^

The recorded variables included the follow-up duration of the studies, number of patients in each study, mean patient's body mass index, mean age of patients, preoperative PSA and prostate volume, clinical Gleason score and TNM stage, pathological Gleason score and TNM stage, operation time, length of hospital stay, perioperative blood loss and transfusion rate, postoperative complications stratified by the Clavien-Dindo classification,^13^ urinary continence, erectile function, catheterization time and nerve-sparing rate. Minor complications were defined as Clavien-Dindo grades I and II, while major complications were defined as Clavien-Dindo grades III and IV. Functional and specific perioperative complications were recorded as dichotomous variables, including rectal damage, anastomotic leakage, lymphocele, lymphedema, stricture, bladder neck contracture, urinary retention, ICU requirement, deep vein thrombosis, ileus and pulmonary embolism. Intraoperative and oncological variables were recorded as continuous variables, including operative time, number of nodes retrieved, number of positive lymph nodes, period with drain, recurrence after surgery, and overall survival.

### Quality assessment and risk of bias

RCTs were appraised with ROB-2, a tool from the Cochrane Collaboration for assessing the risk of bias in randomized trials. Studies were rated as having a high, low, or some concerns risk of bias across five domains: selection, performance, detection, attrition, and reporting biases.^14^ Publication bias was examined using funnel-plot analysis of point estimates according to study weights and Egger's regression test.

### Statistical analysis

This systematic review and meta-analysis was performed in accordance with the Cochrane Collaboration and the Preferred Reporting Items for Systematic Reviews and Meta-Analysis (PRISMA) statement guidelines.^15^ Odds-Ratios (OR) with 95 % Confidence Intervals were used to compare treatment effects for categorical endpoints. Cochran *Q* test and I^2^ statistics were used to assess for heterogeneity; p-values inferior to 0.10 and I^2^ > 25 % were considered significant for heterogeneity. The authorsused a fixed-effect model for outcomes with low heterogeneity (I^2^ < 25 %). Otherwise, a DerSimonian and Laird random-effects model was used. The authorsalso performed a sensitivity analysis with the generic inverse variance method using adjusted risk estimates from observational studies, when available. In addition, a random effect meta-regression analysis was performed to assess the impact of DAPT duration on overall risk ratios. Review Manager 5.4 (Cochrane Centre, The Cochrane Collaboration, Denmark).

## Results

### Literature screening

**Fig. 1:**
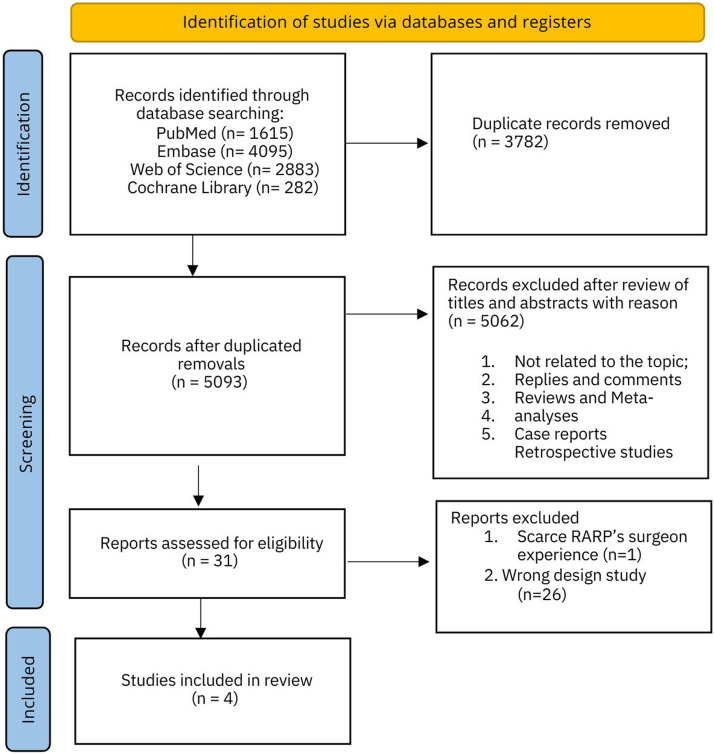
Shows the PRISMA flowchart of the literature search.

A total of 8875 records were identified through the literature search. After automatically removing duplicates, 5092 records remained. Upon reviewing the titles and abstracts, 5062 records were excluded due to not aligning with the study's objectives. Thirty studies were selected for full-text review, but just 4 were RCT and, therefore, were included in the statistical analysis (Fig. 1)

### Quality assessment

During the quality assessment, the study by Coughlin et al.^16^ used strict randomization and allocation methods, although it showed a high missing outcome data (more than 5 %). Still, the study investigators involved in the data analysis and the pathologist who reviewed the biopsy and opened radical prostatectomy specimens were masked to surgical treatment. Nahas et al.^9^ also followed strict randomization and allocation methods, conducting the analysis using the as-treated approach restricting crossovers. The data was collected by a nonblinded institutional nurse who was not linked to the urological department. Therefore, both studies presented the highest quality assessment. Guazzoni et al.^17^ did not present deviations from the intended intervention or missing outcome data. However, he did not discriminate if there was a blinding procedure in the data analysis and just evaluated patients until the fifth post-operatory. None of those studies presented a blinded intervention, increasing the risk of bias.

**Fig. 2:**
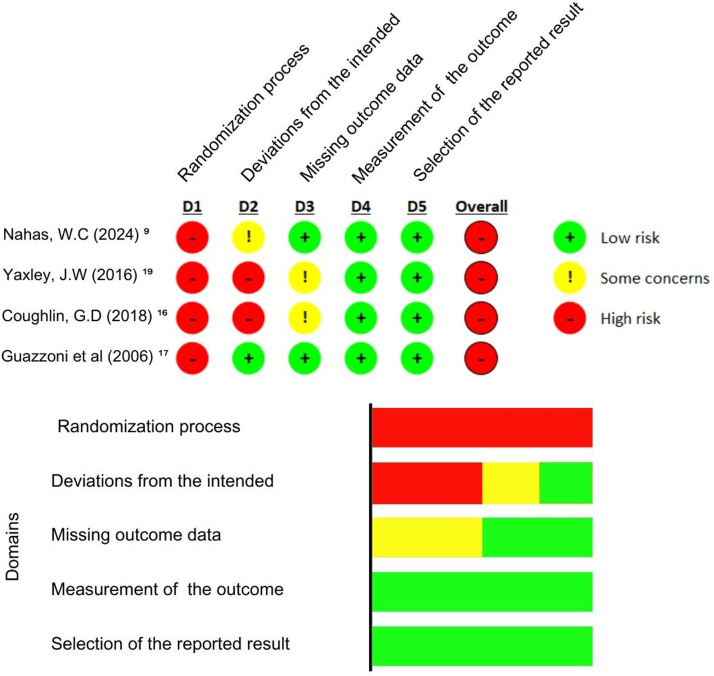
RoB 2.

The risk of bias was evaluated by the Risk of Bias Tool (RoB 2)^14^ (Fig. 2).

### Pooled analysis of all studies

Three randomized controlled trials were included in this analysis. Two of them compare RARP with ORP, and the other one, LP with ORP. The first RCT was published in 2006 by Guazzoni et al.,^17^ and the most recent study, was in July 2024. Yaxley et al.^18^ preceded Coughlin et al.,^16^ describing the early outcomes (3-months) from the same patient group.

Regarding the average age of patients, Guazzoni et al. included patients with a mean age of 62-years, Coughlin et al. with a mean age of 60-years and Nahas et al.^9^^,^^16^, ^17^ with a mean age of 64-years. In Table 1, the authorssummarized baseline data about the selected studies.

**Table 1:** Baseline characteristics of the selected studies.

Name, Year	Trial period	Country	Design	N° of patients	ORP	MIRP	Follow-up	Age (y). mean (SD)		PSA (ng/mL). mean (SD)	
								RRP	MIRP	RRP	MIRP
Nahas, W.C (2024)^9^	Feb 2014 ‒ Jul 2021	Brazil	RCT	327	156	171	36 months	64.0 (3.22)	64.0 (3.16)	7.9 (1.52)	7.2 (1.63)
Coughlin, G.D (2018)^16^	Aug 2010 ‒ Nov 2014	Australia	RCT	296	146	150	24 months	60.38 (5.81)	59.64 (6.63)	7.57 (4.07)	7.41 (4.10)
Yaxley, J.W (2016)^18^	Aug 2010 ‒ Nov 2015	Australia	RCT	308	151	157	12 weeks	60.38 (5.81)	59.64 (6.63)	7.57 (4.07)	7.41 (4.10)
Guazzoni, G. (2006)^17^	Not Reported	Italy	RCT	120	60	60	6 days	62.9 (7.4)	62.29 (8.2)	6.5 (3)	6.9 (2.9)

*Coughlin and Yaxley utilized the same cohort.

### Perioperative outcomes

**Fig. 3:**
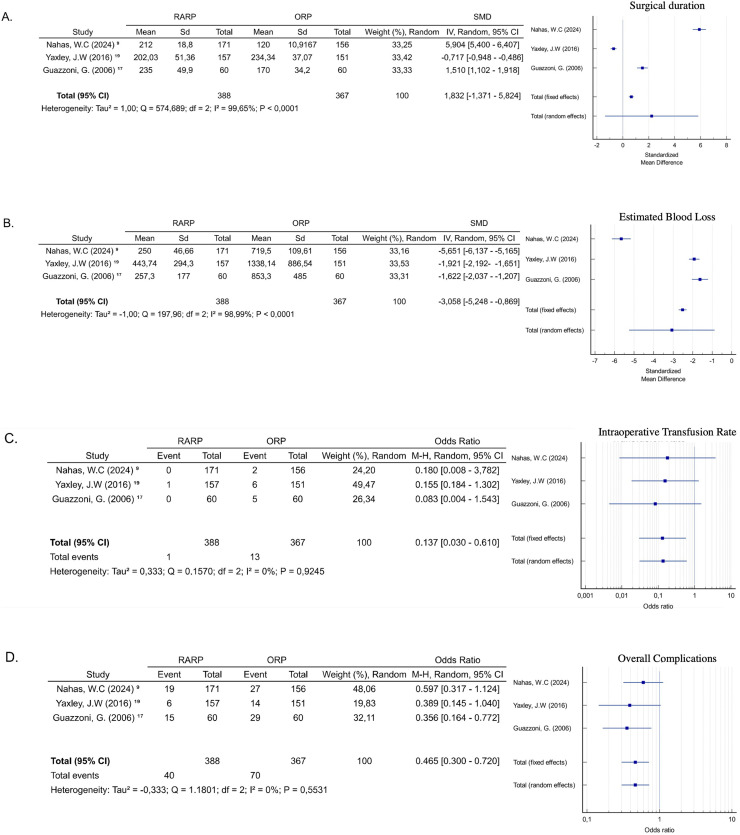

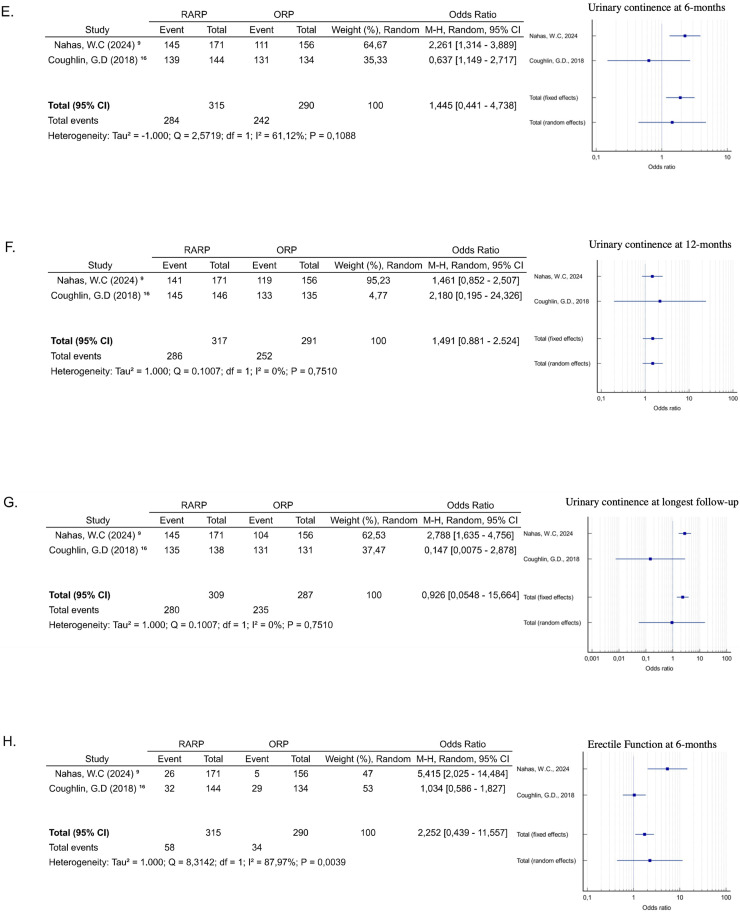

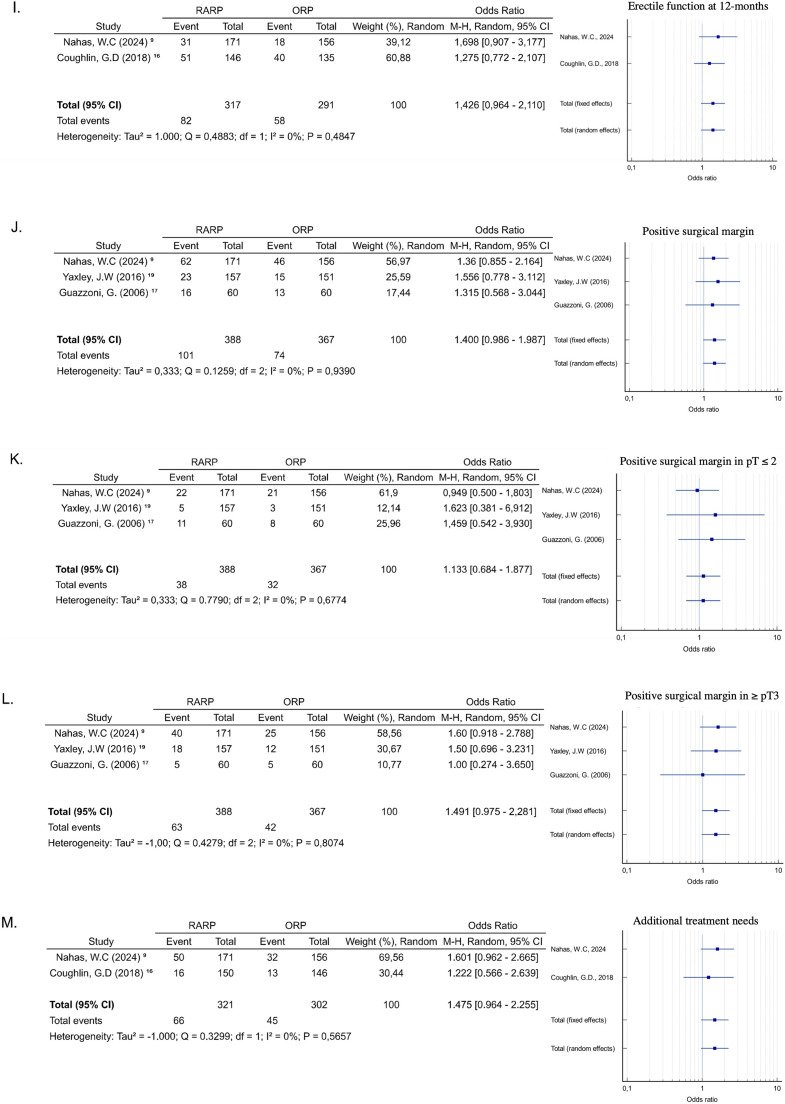
(A) Surgical duration; (B) Estimated blood loss; (C) Intraoperative transfusion rate; (D) Overall Complication; (E) Urinary continence at six months; (F) Urinary continence at 12-months; (G) Urinary continence at longest follow-up; (H) Erectile Function at six months; (I) Erectile function at 12-months; (J) Positive surgical margin; (K) Positive surgical margin in pT ≤2; (L) Positive surgical margin in pT3 and above; (M) Additional treatment.

The three authors cited the surgical time, indicating the difference of time in the ORP group compared to the intervention (RARP and LP) group was not statistically significant (SMD = 1.832; 95 % CI −1.371‒5.824; *p* = 0.225), with high heterogeneity (I^2^ = 99.65 %, *p* < 0.0001) (Fig. 3a). Regarding blood loss, the data showed a lower blood loss in patients undergoing RARP compared to ORP (SMD = −3.058, 95 % CI −5.248 ‒ −0.869; *p* = 0.006). The heterogeneity of the analysis was high (I^2^ = 98.99 %, *p* < 0.0001) (Fig. 3b). All studies reported a lower transfusion rate in patients of the intervention group (RARP and LP) compared to the ORP group, adopting the fixed-effects for meta-analysis (OR = 0.137; 95 % CI 0.030‒0.610; *p* = 0.009), with low heterogeneity (I^2^ = 0 %, *p* = 0.98) (Fig. 3c).

The meta-analysis demonstrated that the intervention group (RARP and LP) has a lower complication rate than the ORP group, with statistical significance (OR = 0.465; 95 % CI 0.30‒0.72; *p* < 0.001) (Fig. 3d). The data showed a low heterogeneity (I^2^ = 0 %, *p* = 0.55). The major complication rates for intervention and ORP group, reported by two studies,^9^^,^^16^ were 1.99 % (6/302 cases) and 4.67 % (15/321), respectively, while the minor complication rates in these studies and groups were 6.29 % (19/302) and 9.03 % (29/321), also respectively.

### Functional outcomes

Urinary continence was defined as using no pads or 0‒1 pads per day. Two studies described post-operative urinary continence.^9^^,^^16^ Analyzing the subgroups according to follow-up, the data also did not show statistical significance. Continence was the same in both groups at 6-months (OR = 1.44, 95 % CI 0.44‒4.73; *p* = 0.54) (Fig. 3e), 12-months (OR = 1.49, 95 % CI 0.88‒2.52; *p* = 0.13) (Fig. 3f), and at the longest follow-up analyzed (OR = 0.92, 95 % CI 0.05‒15.66; *p* = 0.05) (Fig. 3g). The heterogeneity of the analyses at 6 months (I^2^ = 61.12 %, *p* = 0.10) and at the longest follow-up (I^2^ = 73.27 %, *p* = 0.05) was high, while at 12-months (I^2^ = 0 %, *p* = 0.75) it was low.

Erectile function was defined as Sexual Health Inventory for Men (SHIM > 16) or erections firm enough for intercourse more than half the time or almost always. No statistically significant difference was observed in erectile function at 6-months (OR = 2.252, 95 % CI 0.43‒11.55; *p* = 0.33) (Fig. 3h) and 12-months (OR = 1.42, 95 % CI 0.964‒2.11; *p* = 0.076) (Fig. 3i). The heterogeneity reported was respectively high (I^2^ = 87.97 %%, *p* = 0.0039) and low, but with no statistical significance (I^2^ = 0 %, *p* = 0.484).

### Oncological outcomes

The three studies reported data on the surgical margin status at pT2 and ≥ pT3 stages.^9^^,^^16^, ^17^ The overall analysis demonstrated there wasn't any relevant difference in the Positive Surgical Margin (PSM) rate between intervention and control groups (OR = 1.40; 95 % CI 0.986‒1.987, *p* = 0.060) (Fig. 3j), with no substantial heterogeneity and no significance level (I^2^ = 0 %, *p* = 0.9390). Similarly, there was no evidence of disparities in PSM between the groups and the pT2 stage (OR = 1.13; 95 % CI 0.684‒1.877, *p* = 0.629) (Fig. 3k) and ≥ pT3 stages (OR = 1.491; 95 % CI 0.975‒2.281, *p* = 0.065) (Fig. 3l), with low heterogeneity (I^2^ = 0 %, *p* = 0.67 and I^2^ = 0 %, *p* = 0.80, respectively).

Guazzoni et al.,^17^ did not report the follow-up tracking after the surgery, but the other authors did. The meta-analysis of these studies indicated the occurrence of additional treatment of prostate cancer was similar in the groups (OR = 1.47; 95 % CI 0.96‒2.25, *p* = 0.073), with no heterogeneity (I^2^ = 0 %, *p* = 0.565) (Fig. 3m).

## Discussion

The recent RCT published by Nahas et al.^9^ has provided new evidence to aid in the decision-making process between Robotic-Assisted Radical Prostatectomy (RARP) and Open Retropubic Radical Prostatectomy (ORP), marking the second RCT on this topic globally. In this systematic review with meta-analysis, minimally invasive surgeries were associated with lower perioperative blood loss, reduced transfusion rates, and decreased perioperative complication rates. No differences were observed between the groups regarding long-term oncologic and functional outcomes, but these analyses included only two studies since Guazzoni et al.^17^ reported only early and perioperative outcomes.

Due to the lack of RCT studies addressing this topic, there are no systematic reviews with meta-analyses exclusively based on this type of article in the literature. Therefore, the authorscompared these results with systematic reviews that included prospective, non-randomized studies published in 2018, 2019, and 2023.^19^, ^20^, ^21^ Ilic et al.^20^ and Cao et al.^21^ compared open surgeries with robotic-assisted or laparoscopic approaches, while Wang et al.^19^ excluded laparoscopic surgeries.

Operative time was evaluated by Cao et al.^21^ and Wang et al.,^19^ showing a significantly longer duration for RARP/LRP. In the present results, this difference was not observed. Although Nahas et al.^9^ and Guazzoni et al.^17^ demonstrated longer durations for RARP/LRP, Yaxley et al.^18^ reported shorter operative time for the minimally invasive group, likely due to the study being conducted by a single surgeon with extensive experience in robotic surgery, which may have influenced this outcome. Nahas et al. began the study in 2014 and finished in 2018, so that the learning curve in the RARP could have influenced the results.

The findings on perioperative blood loss and transfusion rates are align with previous reviews,^19^, ^20^, ^21^ strengthening this association. This outcome is plausible since the deep pelvic region contains small vascular structures,^22^ and minimally invasive techniques allow for enhanced surgical field visualization, leading to increased precision and improved hemostasis. Additionally, the use of pneumoperitoneum reduces venous bleeding.

The present study associated RARP/LRP with a reduction in overall surgical complication rates. Similar findings were reported by Wang et al.,^19^ who noted reduced rates in the RARP group, although this was not observed in the studies by Ilic et al.^20^ and Cao et al.^21^ It is important to highlight that the reduction in complications may be attributed to the increased stability and precision offered by minimally invasive tools. However, these techniques require a learning curve to achieve optimal outcomes, meaning older data may not capture this advantage fully. This underscores the need for studies involving surgeons with comparable experience in both techniques.

Another interesting advantage of minimally invasive surgery for prostate cancer is the possibility of sharing the surgeon's view on external screens, enhancing understanding and improving this learning curve for residents and fellows.^23^ This capability could have a positive impact on future studies, as it contributes to training and procedural familiarity.

This analysis showed no statistically significant difference between the groups for long-term outcomes, including oncologic and functional results. This analysis included two studies,^9^^,^^16^ as Guazzoni et al.^17^ did not report these data. Furthermore, differences between groups regarding urinary continence, and erectile function remain controversial in the literature.^9^^,^^19^^,^^21^^,^^24^ Regarding urinary continence, Nahas et al.^9^ define it as the use of 0‒1 pads per day, whereas Coughlin et al.^16^ define it as the absence of pad use. This highlights the significant variability in definitions applied across studies, a trend that is prevalent in the literature. Such heterogeneity, alongside inadequate independent assessments using validated questionnaires,^16^ further complicates the comparison of functional outcomes. Additionally, another challenge for such comparisons lies in the multifactorial nature of these outcomes, which may be influenced by factors such as age, comorbidities, and body mass index.^25^ Oncologic outcomes, such as positive surgical margins and additional treatment, also show variability,^26^^,^^27^ suggesting that another possible explanation for these findings is the lack of large-sample RCTs about the topic, resulting in lower-quality evidence.

This systematic review with meta-analysis is the first to include three Randomized Controlled Trials (RCTs). However, the primary limitation is the small number of studies, the heterogeneity in surgeons' experience due to the learning curve, and variations in the definition of urinary incontinence. These sources of bias highlight the challenges of conducting randomized controlled trials in surgical research, but future protocols could address these issues to produce higher-quality evidence. Despite its limitations, this study provides the most reliable evidence to date on early outcomes, although additional randomized trials are needed to assess long-term results. Overall, the findings suggest that minimally invasive techniques may offer a safer alternative to the open approach for retropubic prostatectomies.

## Conclusion

In conclusion, this systematic review with meta-analysis suggests that minimally invasive radical prostatectomy offers advantages over open radical prostatectomy regarding blood loss, transfusion rates, and overall complications. However, further data are needed to clarify long-term outcomes. These techniques remain safe and effective for the treatment of clinically localized prostate cancer, with the choice of approach needing to be individualized for each patient.

## Declaration of competing interest

The authors declare no conflicts of interest.

## Appendix Supplementary materials

Image, application 1
