# Supplementary material for: Minimally invasive radical prostatectomy versus open radical prostatectomy: A systematic review and meta-analysis of randomized control trials
Source: Clinics (Sao Paulo). 2025 Apr 27;80:100636. doi: 10.1016/j.clinsp.2025.100636 (PMC12059318; doi:10.1016/j.clinsp.2025.100636)
Supplement: Supplementary file 1 [file mmc1.docx]

**Operative Time**


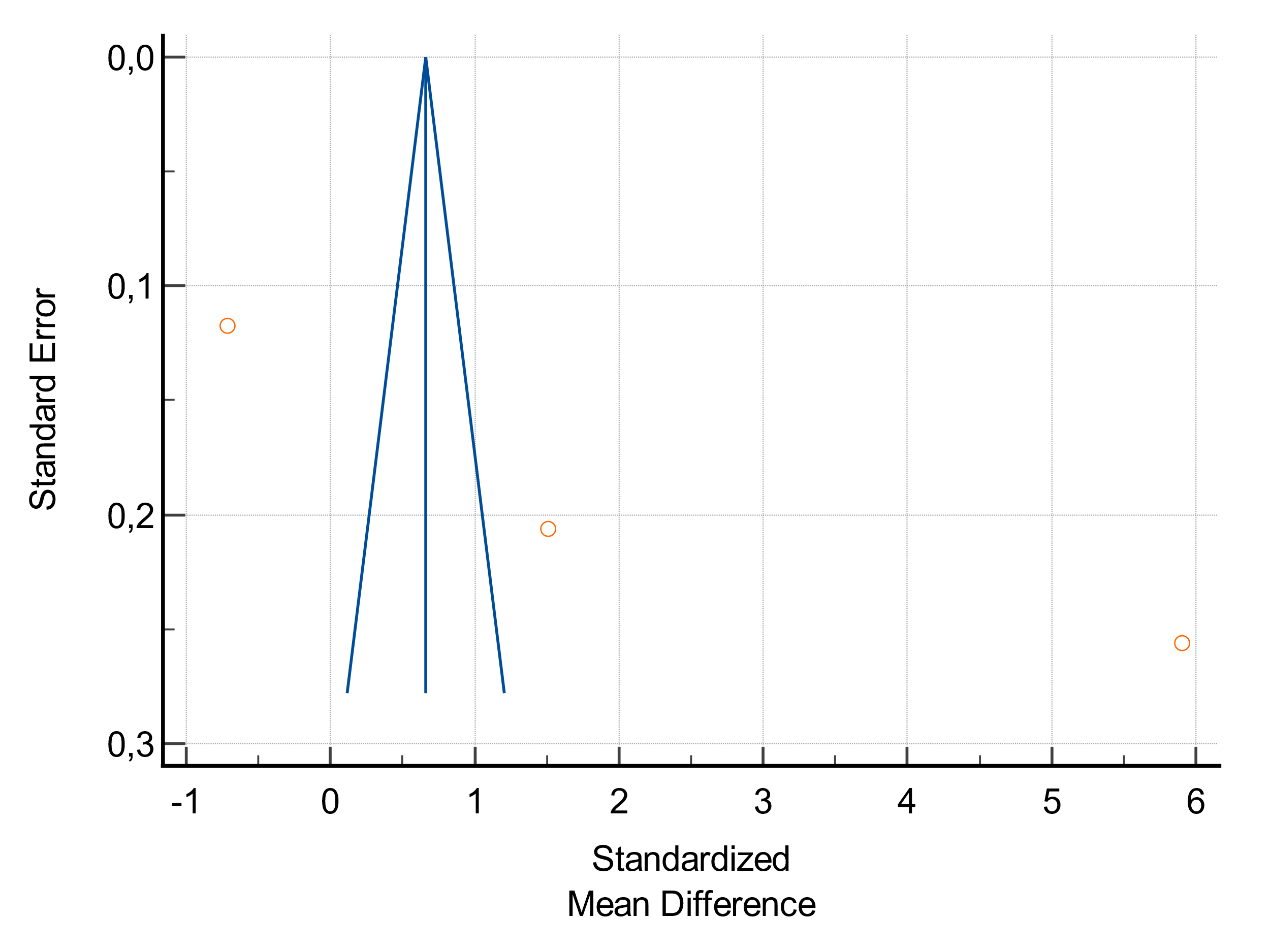


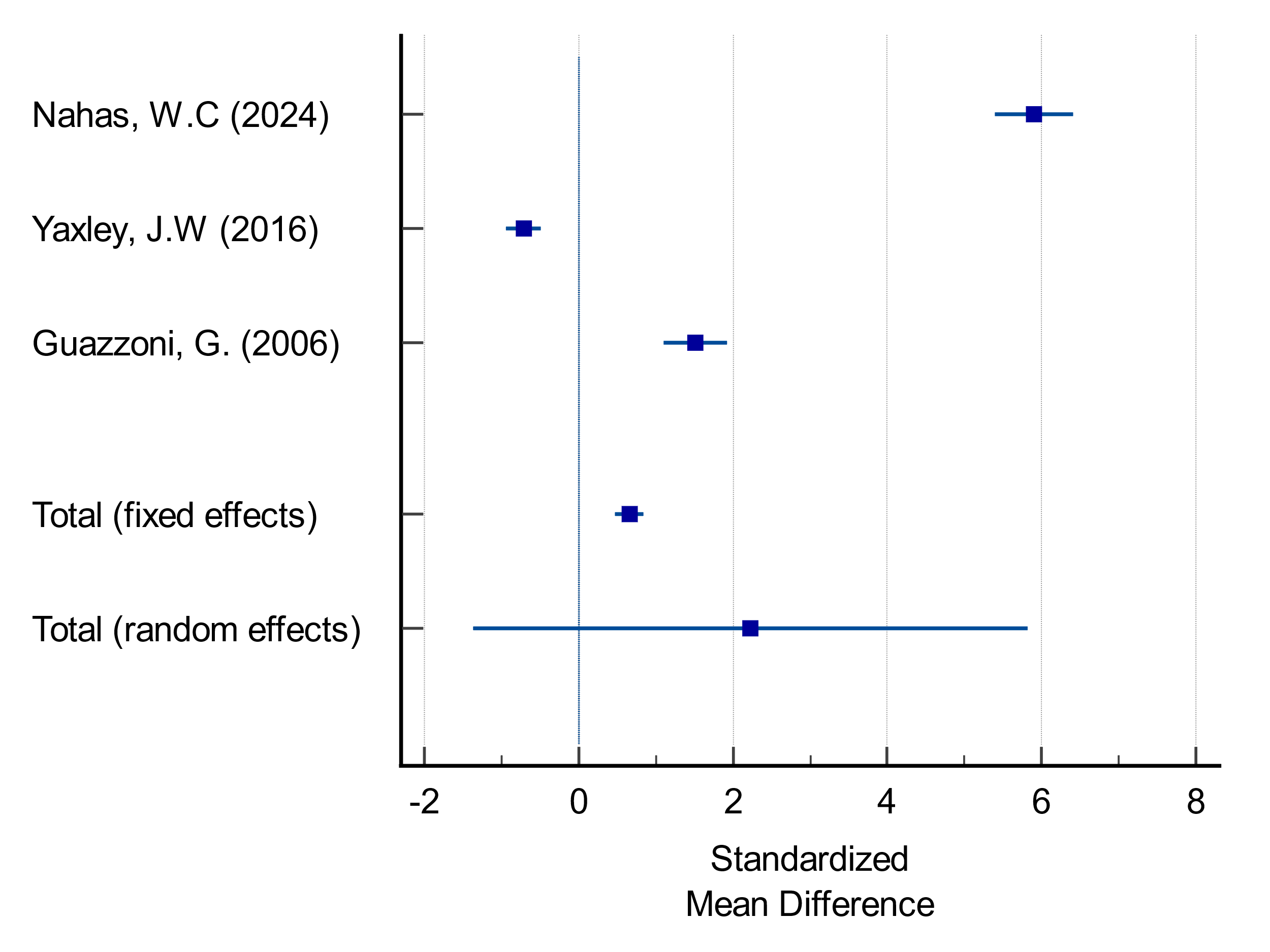


**Meta-analysis: continuous measure**

| Variable for studies | Artigo |
| --- | --- |
| 1. Intervention groups | |
| Variable for number of cases | total_MIRP |
| Variable for mean | op_time_MIRP |
| Variable for SD | op_time_MIRP_mean |
| 2. Control groups | |
| Variable for number of cases | total_ORP |
| Variable for mean | op_time_ORP |
| Variable for SD | op_time_ORP_mean |

| Study | N1 | N2 | Total | SMD | SE | 95% CI | t | P | Weight (%) | |
| --- | --- | --- | --- | --- | --- | --- | --- | --- | --- | --- |
|  |  |  |  |  |  |  |  |  | Fixed | Random |
| Nahas, W.C (2024) | 171 | 156 | 327 | 5,904 | 0,256 | 5,400 to 6,407 |  |  | 13,69 | 33,25 |
| Yaxley, J.W (2016) | 157 | 151 | 308 | -0,717 | 0,117 | -0,948 to -0,486 |  |  | 65,16 | 33,42 |
| Guazzoni, G. (2006) | 60 | 60 | 120 | 1,510 | 0,206 | 1,102 to 1,918 |  |  | 21,15 | 33,33 |
| Total (fixed effects) | 388 | 367 | 755 | 0,660 | 0,0947 | 0,474 to 0,846 | 6,972 | <0,001 | 100,00 | 100,00 |
| Total (random effects) | 388 | 367 | 755 | 2,227 | 1,832 | -1,371 to 5,824 | 1,215 | 0,225 | 100,00 | 100,00 |

**Test for heterogeneity**

| Q | 574,6892 |
| --- | --- |
| DF | 2 |
| Significance level | P < 0,0001 |
| I^2^ (inconsistency) | 99,65% |
| 95% CI for I^2^ | 99,51 to 99,75 |

**Publication bias**

| Egger's test | |
| --- | --- |
| Intercept | 40,2429 |
| 95% CI | -128,9268 to 209,4127 |
| Significance level | P = 0,2034 |
| Begg's test | |
| Kendall's Tau | 1,0000 |
| Significance level | P = 0,1172 |

**Blood Loss**


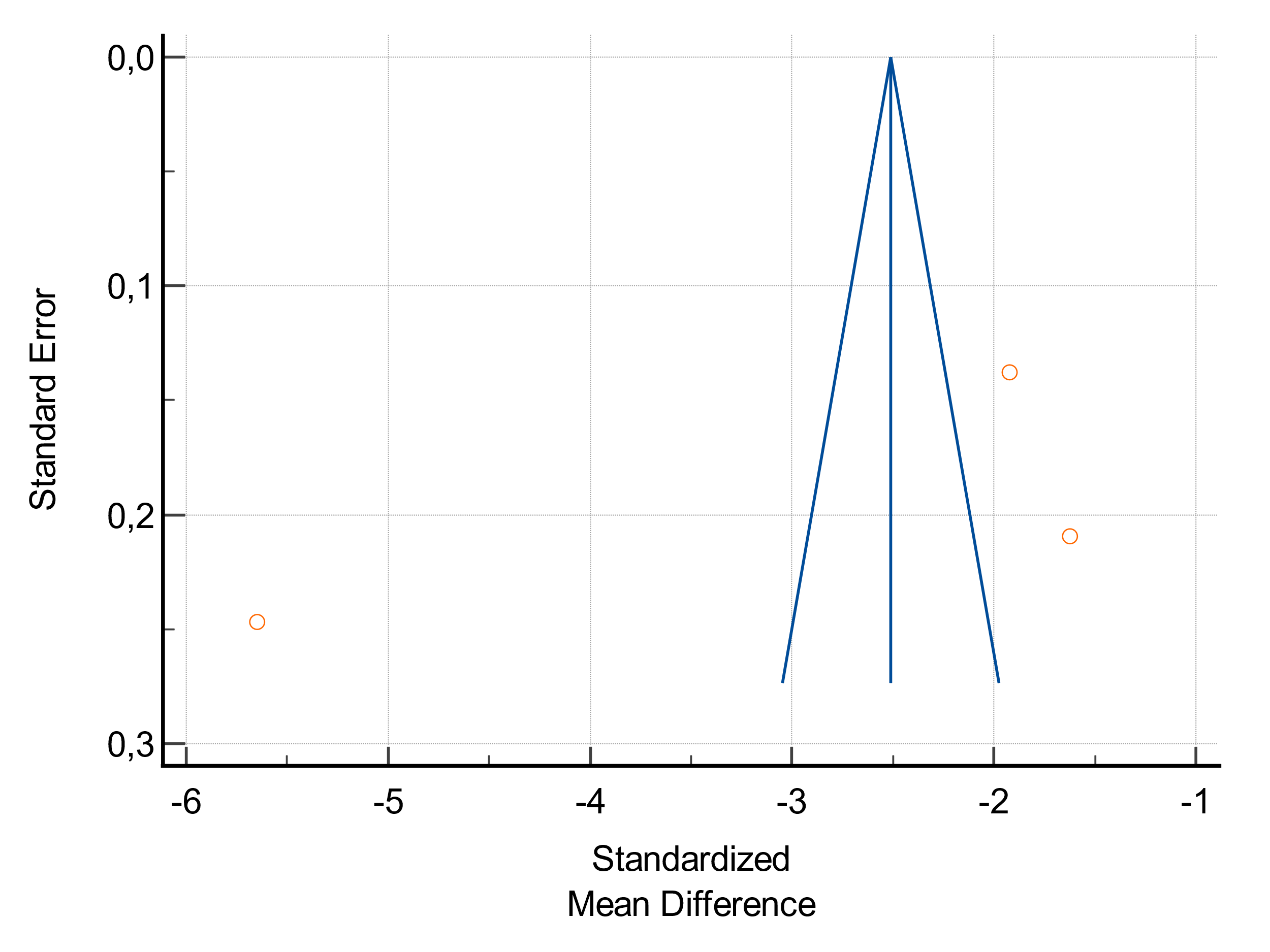


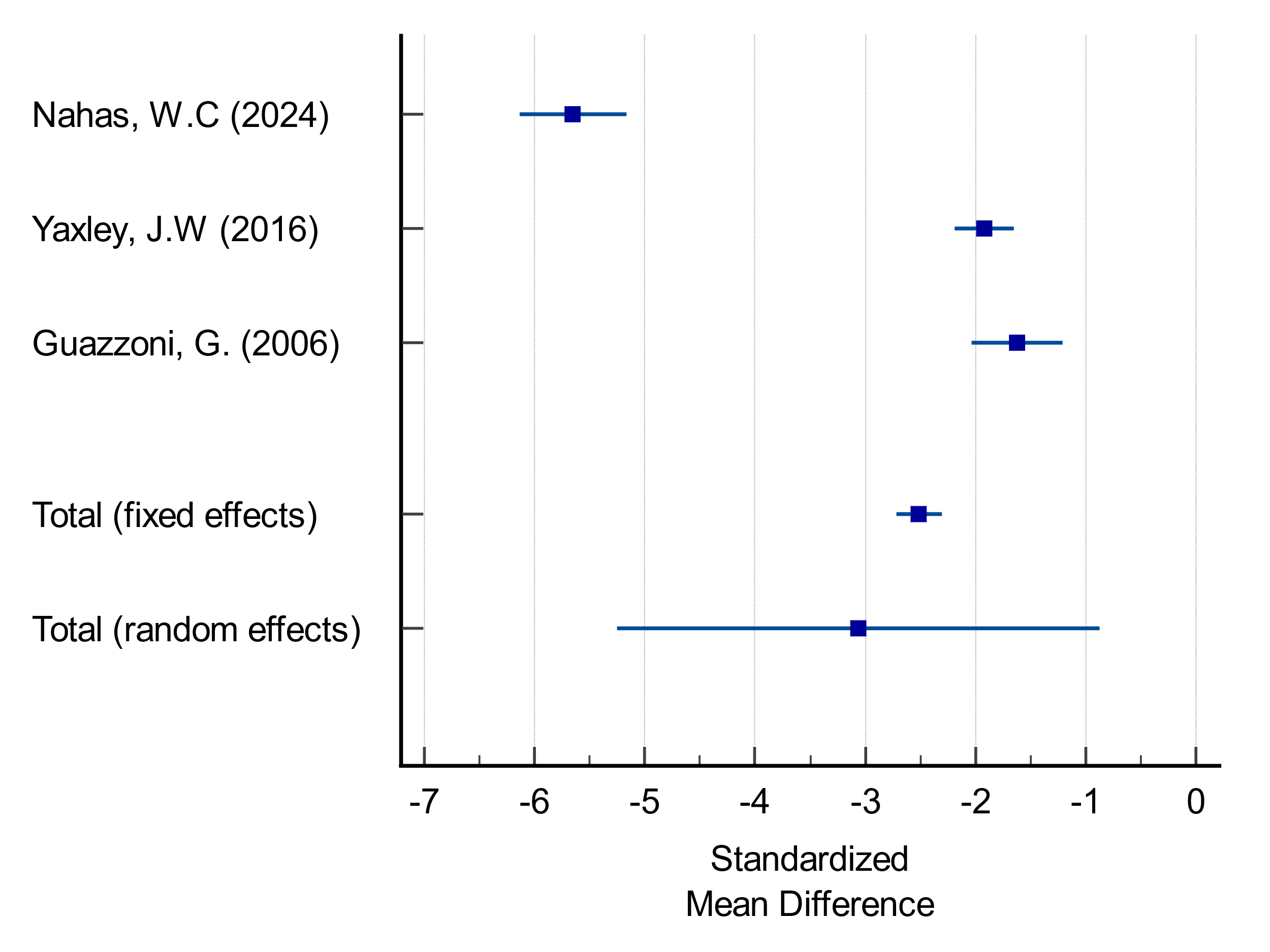


**Meta-analysis: continuous measure**

| Variable for studies | Artigo |
| --- | --- |
| 1. Intervention groups | |
| Variable for number of cases | total_MIRP |
| Variable for mean | bloodloss_MIRP |
| Variable for SD | bloodloss_MIRP_mean |
| 2. Control groups | |
| Variable for number of cases | total_ORP |
| Variable for mean | bloodloss_ORP |
| Variable for SD | bloodloss_ORP_mean |

| Study | N1 | N2 | Total | SMD | SE | 95% CI | t | P | Weight (%) | |
| --- | --- | --- | --- | --- | --- | --- | --- | --- | --- | --- |
|  |  |  |  |  |  |  |  |  | Fixed | Random |
| Nahas, W.C (2024) | 171 | 156 | 327 | -5,651 | 0,247 | -6,137 to -5,165 |  |  | 17,80 | 33,16 |
| Yaxley, J.W (2016) | 157 | 151 | 308 | -1,921 | 0,138 | -2,192 to -1,651 |  |  | 57,43 | 33,53 |
| Guazzoni, G. (2006) | 60 | 60 | 120 | -1,622 | 0,209 | -2,037 to -1,207 |  |  | 24,77 | 33,31 |
| Total (fixed effects) | 388 | 367 | 755 | -2,511 | 0,104 | -2,716 to -2,307 | -24,091 | <0,001 | 100,00 | 100,00 |
| Total (random effects) | 388 | 367 | 755 | -3,058 | 1,115 | -5,248 to -0,869 | -2,743 | 0,006 | 100,00 | 100,00 |

**Test for heterogeneity**

| Q | 197,9649 |
| --- | --- |
| DF | 2 |
| Significance level | P < 0,0001 |
| I^2^ (inconsistency) | 98,99% |
| 95% CI for I^2^ | 98,34 to 99,38 |

**Publication bias**

| Egger's test | |
| --- | --- |
| Intercept | -22,4240 |
| 95% CI | -323,1280 to 278,2801 |
| Significance level | P = 0,5172 |
| Begg's test | |
| Kendall's Tau | -1,0000 |
| Significance level | P = 0,1172 |

**Positive Surgical Margin**


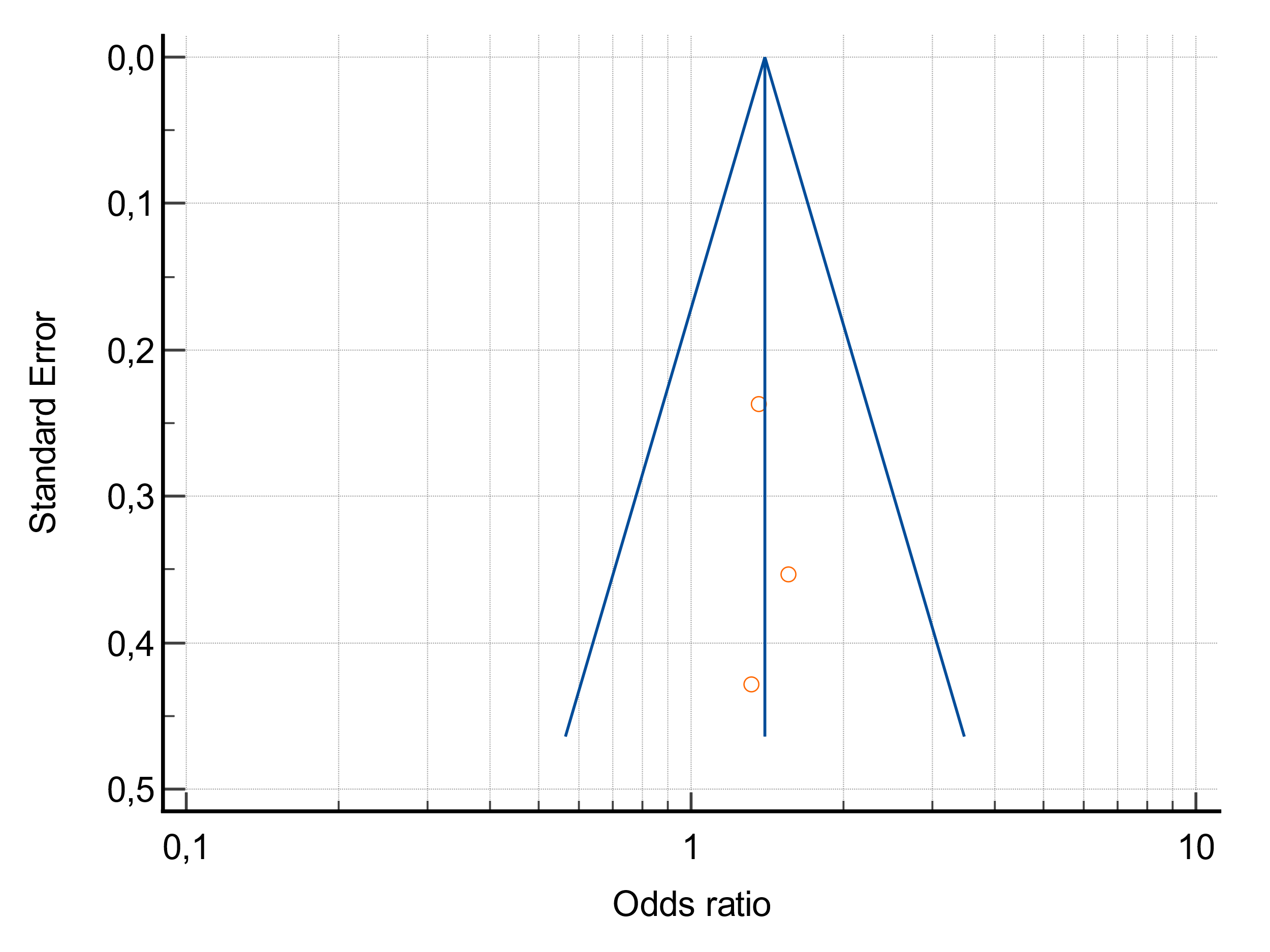


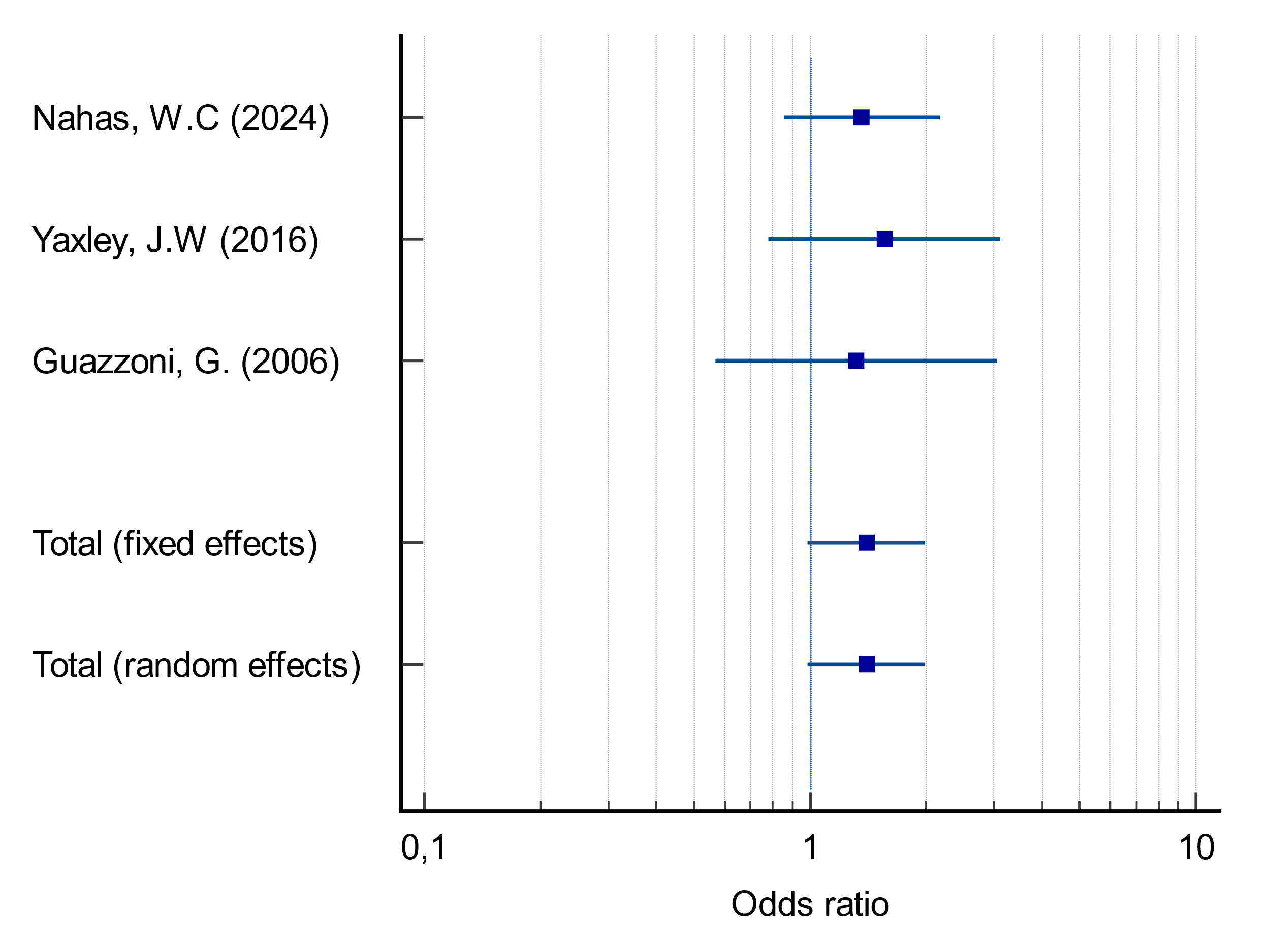


**Meta-analysis: odds ratio**

| Variable for studies | Artigo |
| --- | --- |
| 1. Intervention groups | |
| Variable for total number of cases | total_MIRP |
| Variable for number of positive cases | psm_MIRP |
| 2. Control groups | |
| Variable for total number of cases | total_ORP |
| Variable for number of positive cases | psm_ORP |

| Study | Intervention | Controls | Odds ratio | 95% CI | z | P | Weight (%) | |
| --- | --- | --- | --- | --- | --- | --- | --- | --- |
|  |  |  |  |  |  |  | Fixed | Random |
| Nahas, W.C (2024) | 62/171 | 46/156 | 1,360 | 0,855 to 2,164 |  |  | 56,97 | 56,97 |
| Yaxley, J.W (2016) | 23/157 | 15/151 | 1,556 | 0,778 to 3,112 |  |  | 25,59 | 25,59 |
| Guazzoni, G. (2006) | 16/60 | 13/60 | 1,315 | 0,568 to 3,044 |  |  | 17,44 | 17,44 |
| Total (fixed effects) | 101/388 | 74/367 | 1,400 | 0,986 to 1,987 | 1,883 | 0,060 | 100,00 | 100,00 |
| Total (random effects) | 101/388 | 74/367 | 1,400 | 0,986 to 1,987 | 1,880 | 0,060 | 100,00 | 100,00 |

**Test for heterogeneity**

| Q | 0,1259 |
| --- | --- |
| DF | 2 |
| Significance level | P = 0,9390 |
| I^2^ (inconsistency) | 0,00% |
| 95% CI for I^2^ | 0,00 to 46,72 |

**Publication bias**

| Egger's test | |
| --- | --- |
| Intercept | 0,1838 |
| 95% CI | -10,0798 to 10,4474 |
| Significance level | P = 0,8575 |
| Begg's test | |
| Kendall's Tau | 0,3333 |
| Significance level | P = 0,6015 |

**Overall Complications**


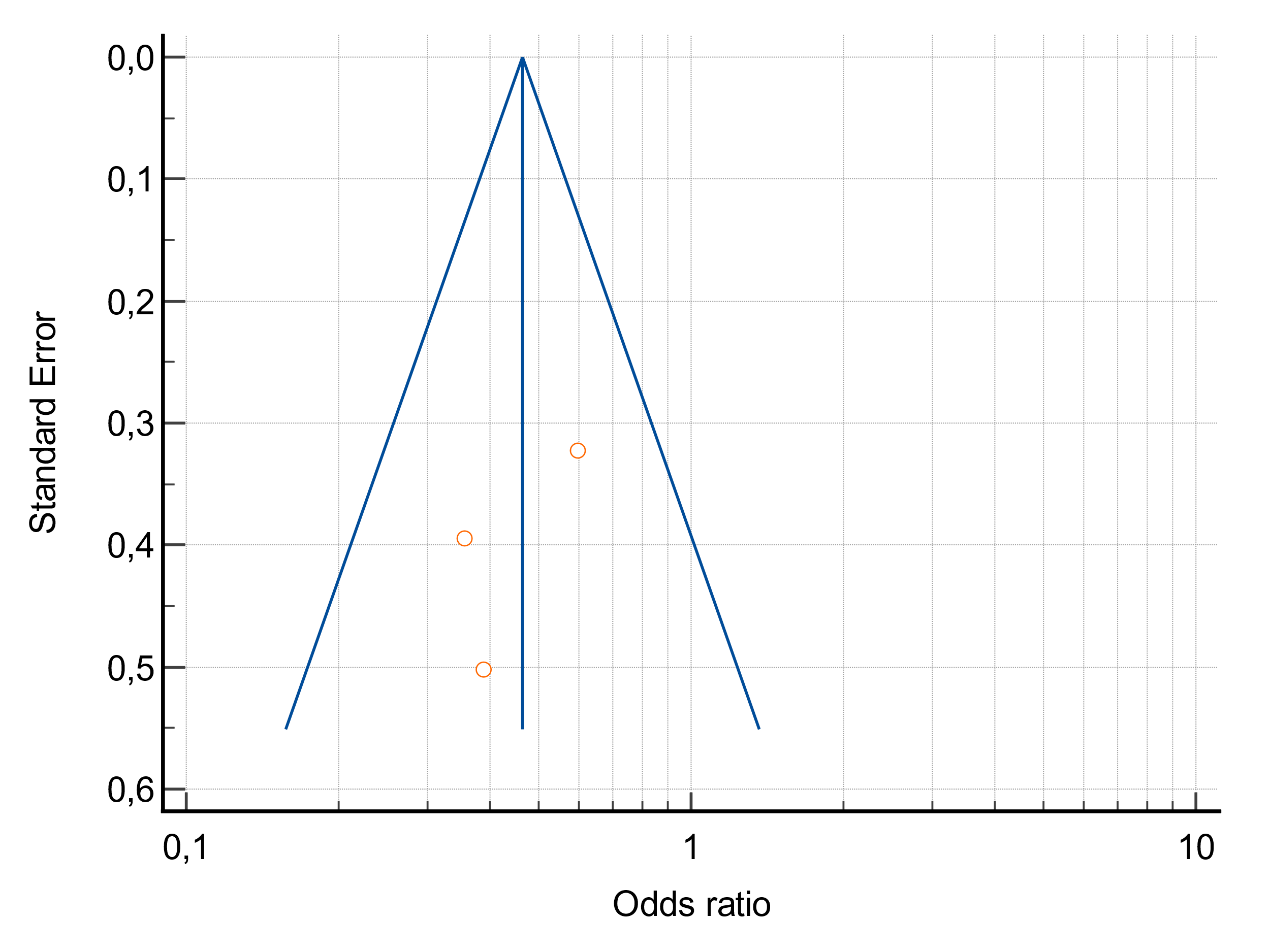


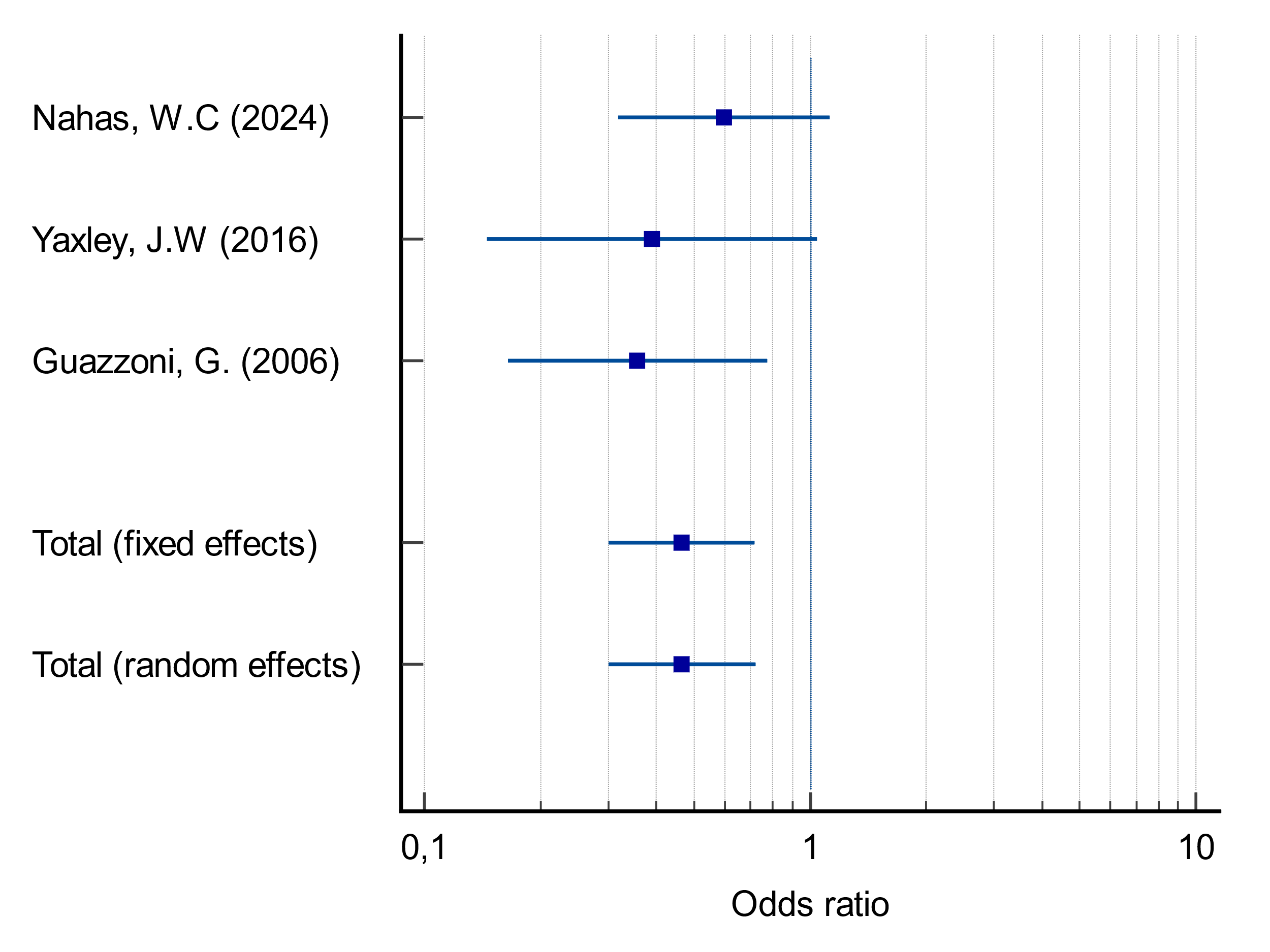


| Variable for studies | Artigo |
| --- | --- |
| 1. Intervention groups | |
| Variable for total number of cases | total_MIRP |
| Variable for number of positive cases | complic_MIRP |
| 2. Control groups | |
| Variable for total number of cases | total_ORP |
| Variable for number of positive cases | complic_ORP |

| Study | Intervention | Controls | Odds ratio | 95% CI | z | P | Weight (%) | |
| --- | --- | --- | --- | --- | --- | --- | --- | --- |
|  |  |  |  |  |  |  | Fixed | Random |
| Nahas, W.C (2024) | 19/171 | 27/156 | 0,597 | 0,317 to 1,124 |  |  | 48,06 | 48,06 |
| Yaxley, J.W (2016) | 6/157 | 14/151 | 0,389 | 0,145 to 1,040 |  |  | 19,83 | 19,83 |
| Guazzoni, G. (2006) | 15/60 | 29/60 | 0,356 | 0,164 to 0,772 |  |  | 32,11 | 32,11 |
| Total (fixed effects) | 40/388 | 70/367 | 0,464 | 0,300 to 0,717 | -3,454 | 0,001 | 100,00 | 100,00 |
| Total (random effects) | 40/388 | 70/367 | 0,465 | 0,300 to 0,720 | -3,428 | 0,001 | 100,00 | 100,00 |

**Test for heterogeneity**

| Q | 1,1846 |
| --- | --- |
| DF | 2 |
| Significance level | P = 0,5531 |
| I^2^ (inconsistency) | 0,00% |
| 95% CI for I^2^ | 0,00 to 94,34 |

**Publication bias**

| Egger's test | |
| --- | --- |
| Intercept | -2,7092 |
| 95% CI | -32,5289 to 27,1105 |
| Significance level | P = 0,4545 |
| Begg's test | |
| Kendall's Tau | -0,3333 |
| Significance level | P = 0,6015 |

**Transfusion Rate**


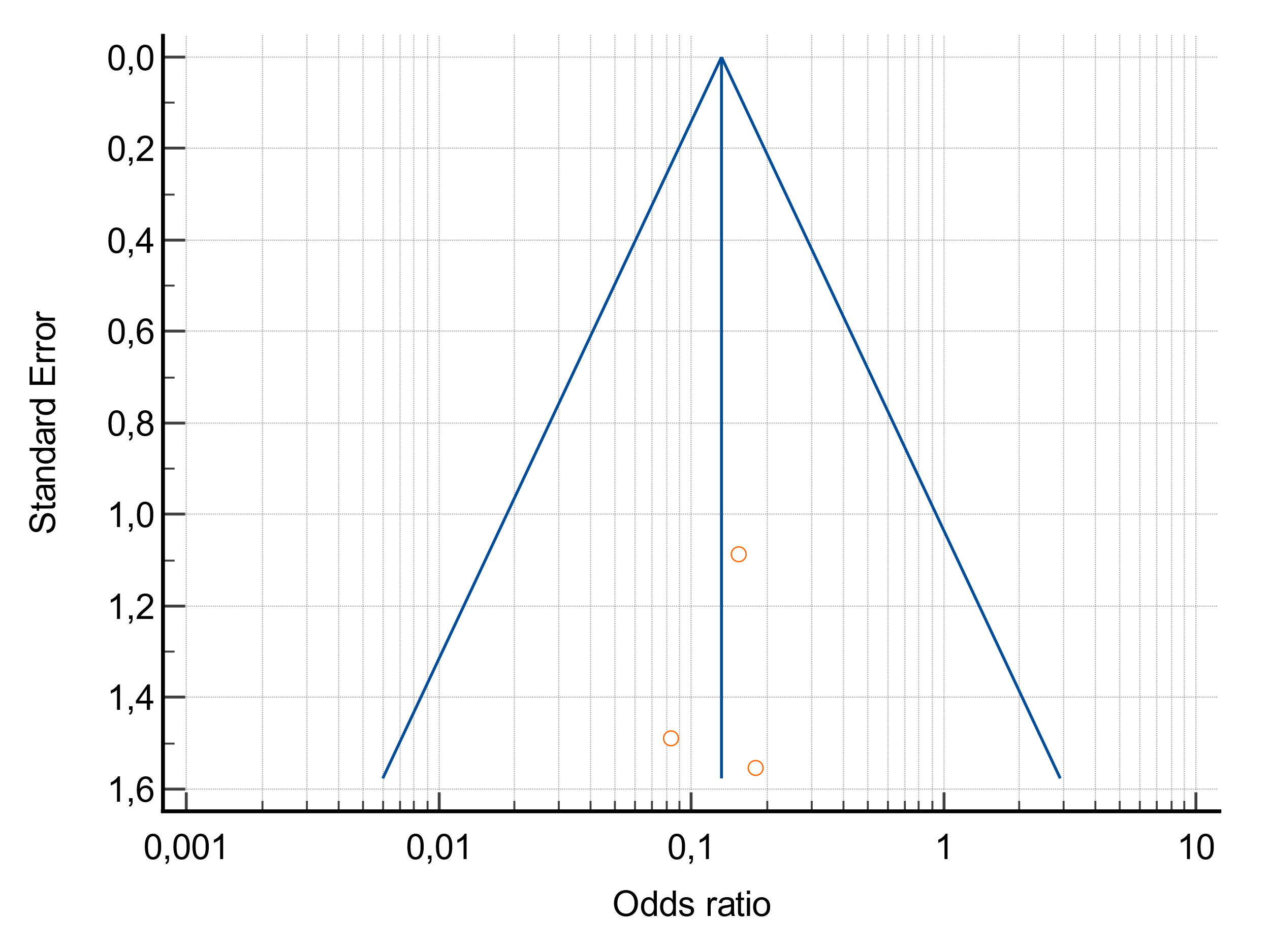


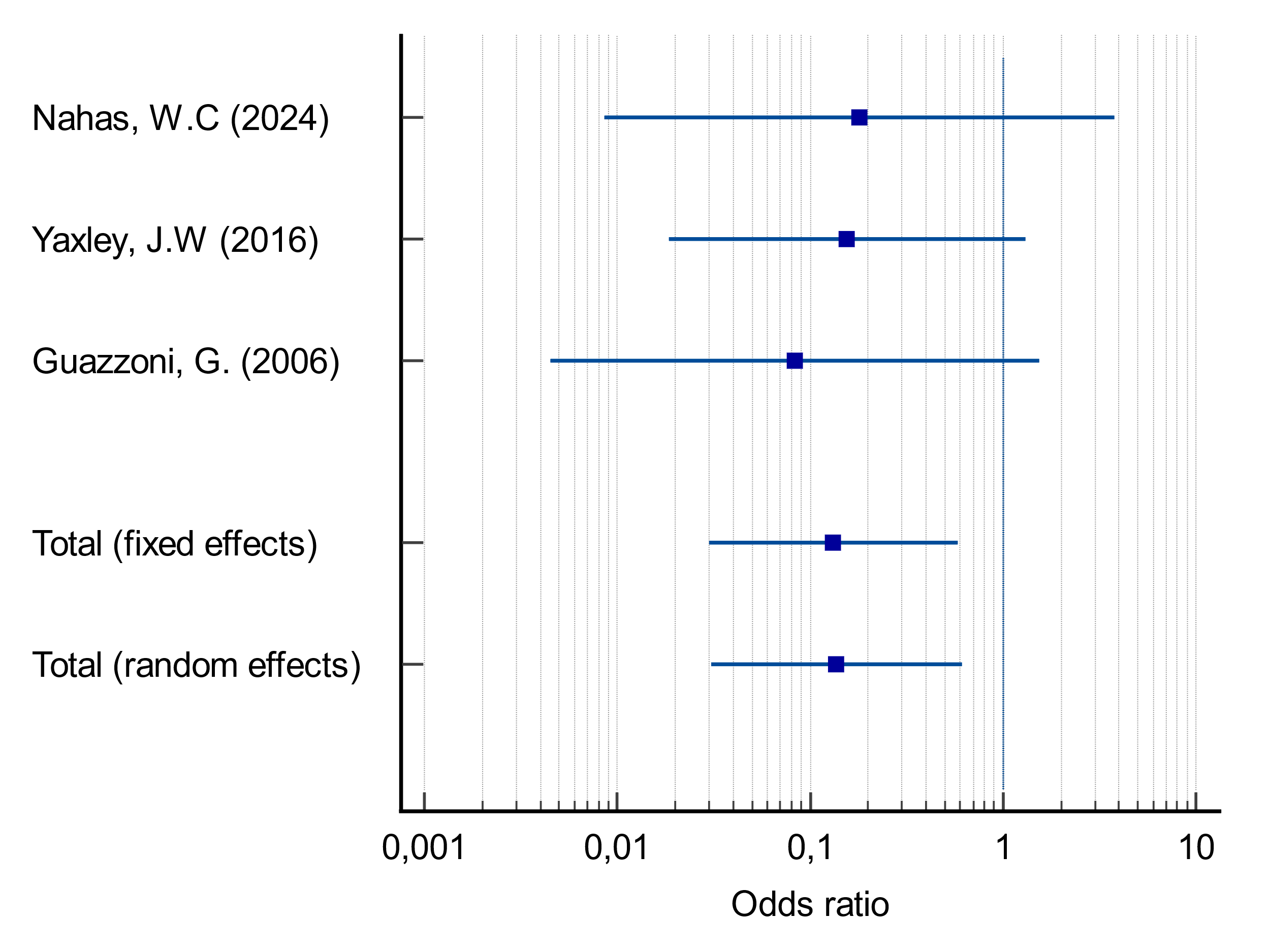


**Meta-analysis: odds ratio**

| Variable for studies | Artigo |
| --- | --- |
| 1. Intervention groups | |
| Variable for total number of cases | total_MIRP |
| Variable for number of positive cases | transf_MIRP |
| 2. Control groups | |
| Variable for total number of cases | total_ORP |
| Variable for number of positive cases | transf_ORP |

| Study | Intervention | Controls | Odds ratio | 95% CI | z | P | Weight (%) | |
| --- | --- | --- | --- | --- | --- | --- | --- | --- |
|  |  |  |  |  |  |  | Fixed | Random |
| Nahas, W.C (2024) | 0/171 | 2/156 | 0,180 | 0,00858 to 3,782 |  |  | 24,20 | 24,20 |
| Yaxley, J.W (2016) | 1/157 | 6/151 | 0,155 | 0,0184 to 1,302 |  |  | 49,47 | 49,47 |
| Guazzoni, G. (2006) | 0/60 | 5/60 | 0,0834 | 0,00451 to 1,543 |  |  | 26,34 | 26,34 |
| Total (fixed effects) | 1/388 | 13/367 | 0,132 | 0,0298 to 0,585 | -2,665 | 0,008 | 100,00 | 100,00 |
| Total (random effects) | 1/388 | 13/367 | 0,137 | 0,0305 to 0,610 | -2,607 | 0,009 | 100,00 | 100,00 |

**Test for heterogeneity**

| Q | 0,1570 |
| --- | --- |
| DF | 2 |
| Significance level | P = 0,9245 |
| I^2^ (inconsistency) | 0,00% |
| 95% CI for I^2^ | 0,00 to 57,27 |

**Publication bias**

| Egger's test | |
| --- | --- |
| Intercept | -0,4397 |
| 95% CI | -17,0731 to 16,1937 |
| Significance level | P = 0,7937 |
| Begg's test | |
| Kendall's Tau | 0,3333 |
| Significance level | P = 0,6015 |

**Additional Treatment**


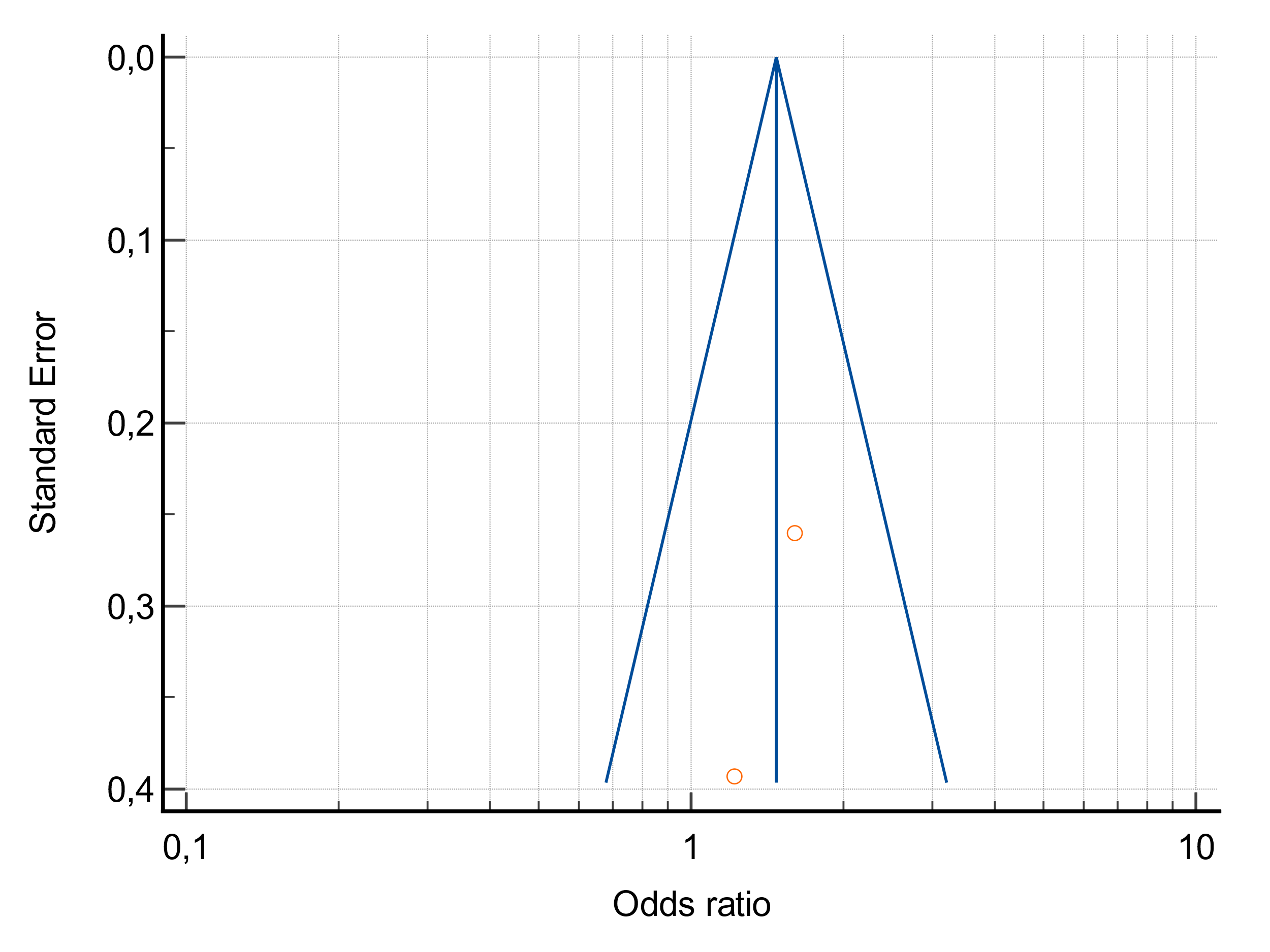


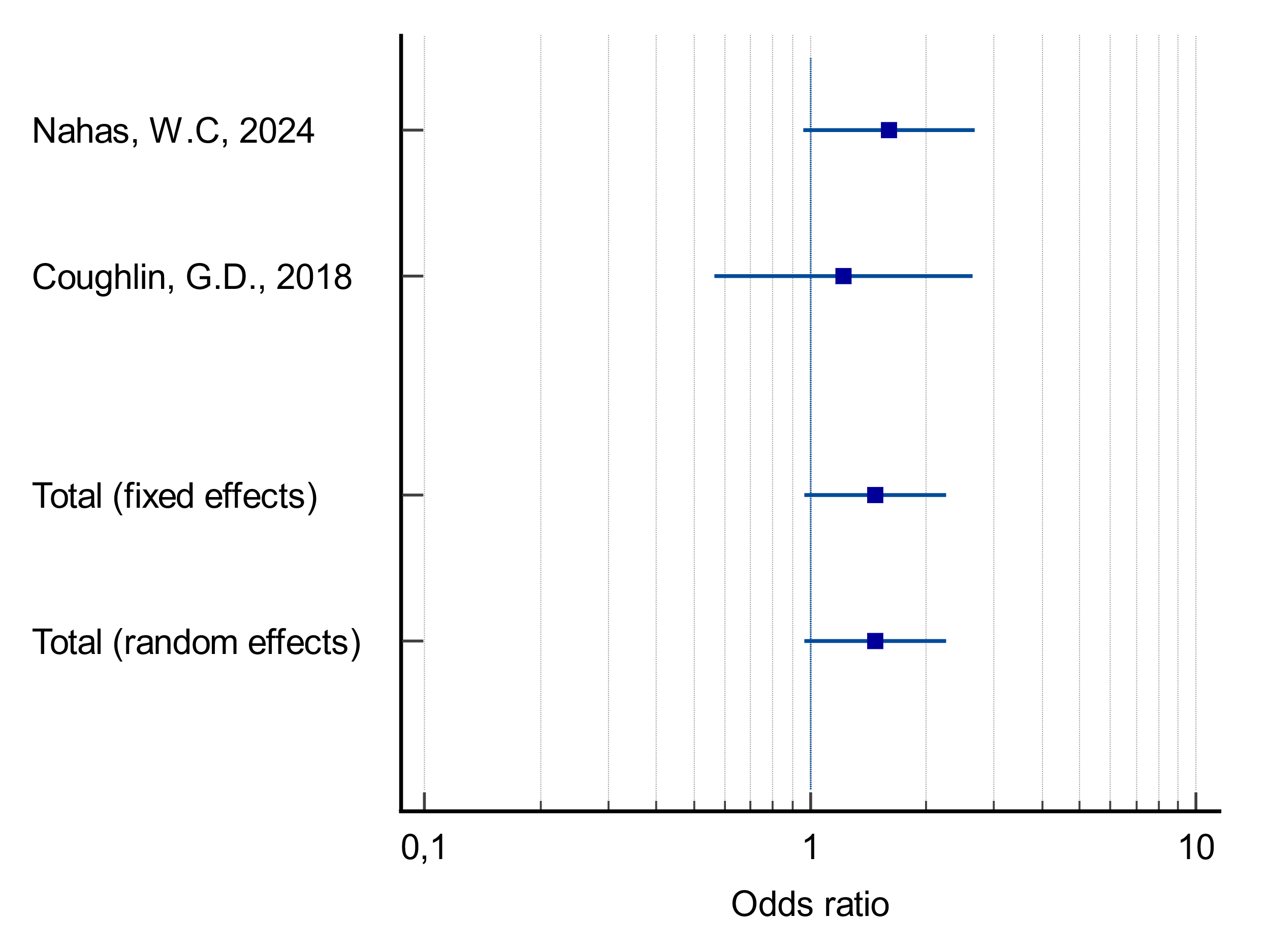


| Variable for studies | Study |
| --- | --- |
| 1. Intervention groups | |
| Variable for total number of cases | MIRP_total MIRP total |
| Variable for number of positive cases | tratamento_adicional_MIRP |
| 2. Control groups | |
| Variable for total number of cases | Open_total Open _total |
| Variable for number of positive cases | tratamento_adicional_Open |

| Study | Intervention | Controls | Odds ratio | 95% CI | z | P | Weight (%) | |
| --- | --- | --- | --- | --- | --- | --- | --- | --- |
|  |  |  |  |  |  |  | Fixed | Random |
| Nahas, W.C, 2024 | 50/171 | 32/156 | 1,601 | 0,962 to 2,665 |  |  | 69,56 | 69,56 |
| Coughlin, G.D., 2018 | 16/150 | 13/146 | 1,222 | 0,566 to 2,639 |  |  | 30,44 | 30,44 |
| Total (fixed effects) | 66/321 | 45/302 | 1,475 | 0,965 to 2,255 | 1,795 | 0,073 | 100,00 | 100,00 |
| Total (random effects) | 66/321 | 45/302 | 1,475 | 0,964 to 2,255 | 1,791 | 0,073 | 100,00 | 100,00 |

**Test for heterogeneity**

| Q | 0,3299 |
| --- | --- |
| DF | 1 |
| Significance level | P = 0,5657 |
| I^2^ (inconsistency) | 0,00% |
| 95% CI for I^2^ | 0,00 to 0,00 |

**Publication bias**

| Egger's test | |
| --- | --- |
| Intercept | -2,0351 |
| 95% CI |  |
| Significance level | P < 0,0001 |
| Begg's test | |
| Kendall's Tau | -1,0000 |
| Significance level | P = 0,3173 |

**Positive surgical margin rate pT ≤ 2**


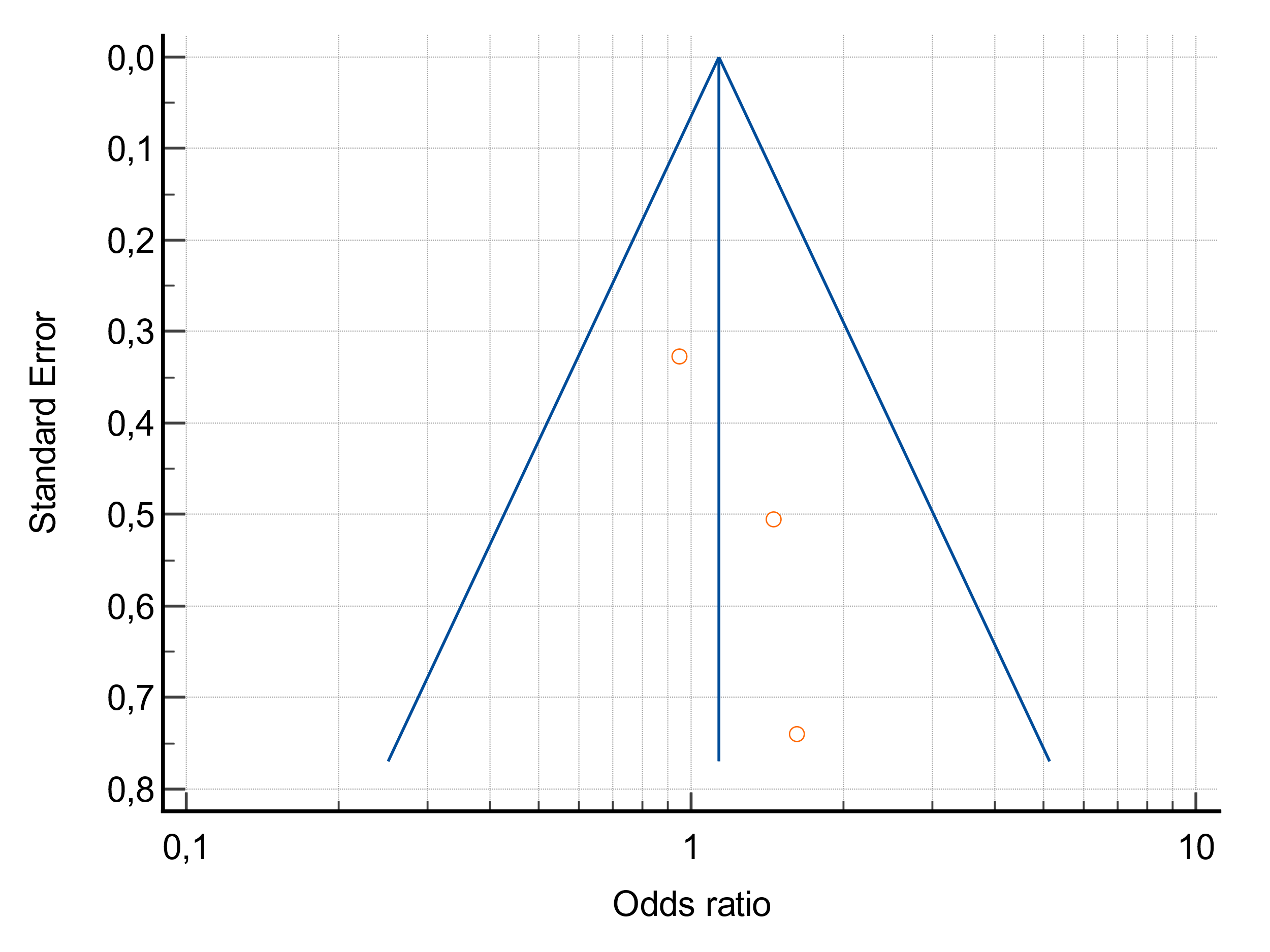


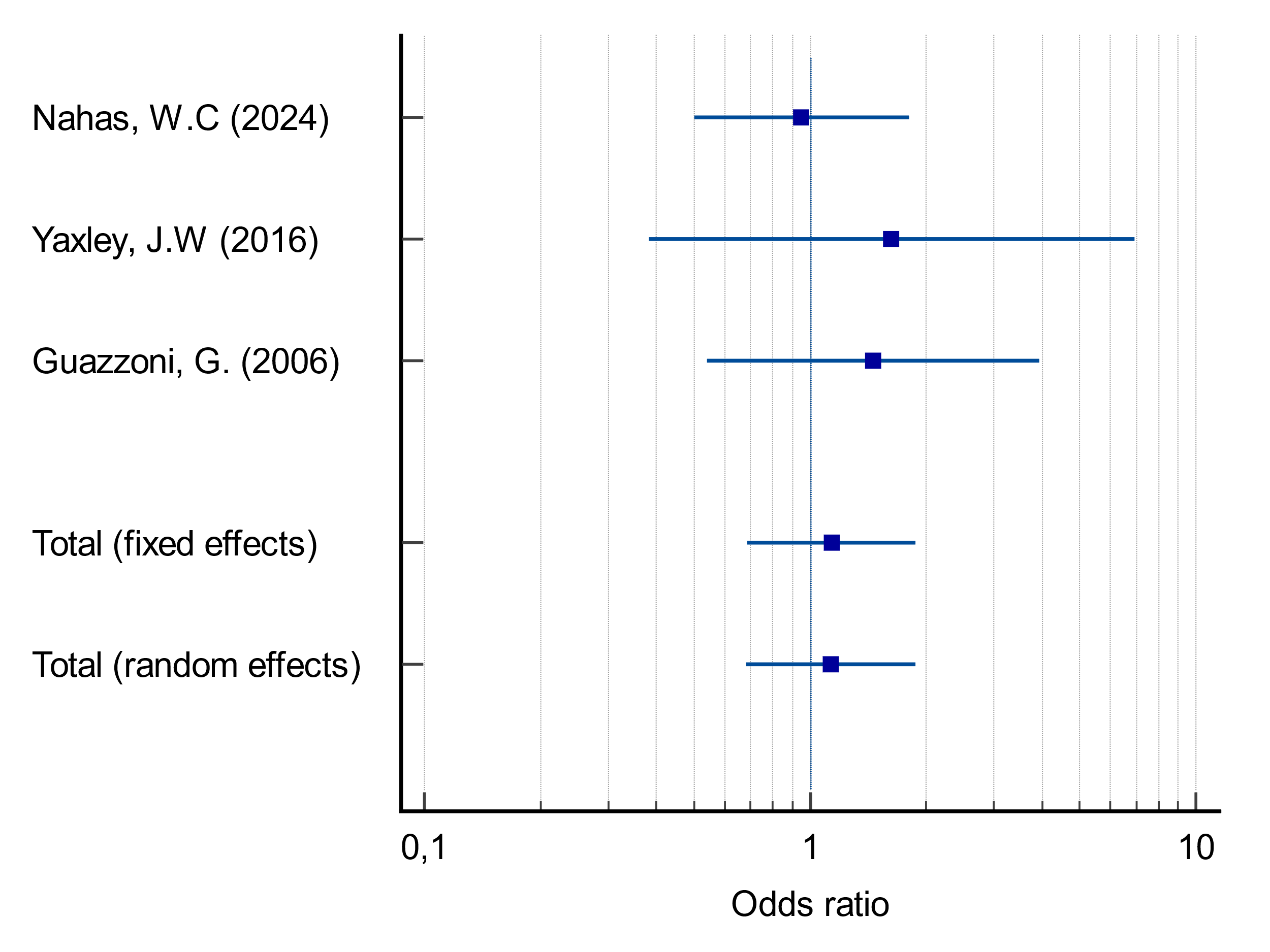


**Meta-analysis: odds ratio**

| Variable for studies | Artigo |
| --- | --- |
| 1. Intervention groups | |
| Variable for total number of cases | total_MIRP |
| Variable for number of positive cases | psmt2_MIRP |
| 2. Control groups | |
| Variable for total number of cases | total_ORP |
| Variable for number of positive cases | psmt2_ORP |

| Study | Intervention | Controls | Odds ratio | 95% CI | z | P | Weight (%) | |
| --- | --- | --- | --- | --- | --- | --- | --- | --- |
|  |  |  |  |  |  |  | Fixed | Random |
| Nahas, W.C (2024) | 22/171 | 21/156 | 0,949 | 0,500 to 1,803 |  |  | 61,90 | 61,90 |
| Yaxley, J.W (2016) | 5/157 | 3/151 | 1,623 | 0,381 to 6,912 |  |  | 12,14 | 12,14 |
| Guazzoni, G. (2006) | 11/60 | 8/60 | 1,459 | 0,542 to 3,930 |  |  | 25,96 | 25,96 |
| Total (fixed effects) | 38/388 | 32/367 | 1,135 | 0,687 to 1,876 | 0,495 | 0,621 | 100,00 | 100,00 |
| Total (random effects) | 38/388 | 32/367 | 1,133 | 0,684 to 1,877 | 0,484 | 0,629 | 100,00 | 100,00 |

**Test for heterogeneity**

| Q | 0,7790 |
| --- | --- |
| DF | 2 |
| Significance level | P = 0,6774 |
| I^2^ (inconsistency) | 0,00% |
| 95% CI for I^2^ | 0,00 to 91,39 |

**Publication bias**

| Egger's test | |
| --- | --- |
| Intercept | 1,5170 |
| 95% CI | -5,8845 to 8,9186 |
| Significance level | P = 0,2334 |
| Begg's test | |
| Kendall's Tau | 0,3333 |
| Significance level | P = 0,6015 |

**Positive surgical margin rate pT3 and above**


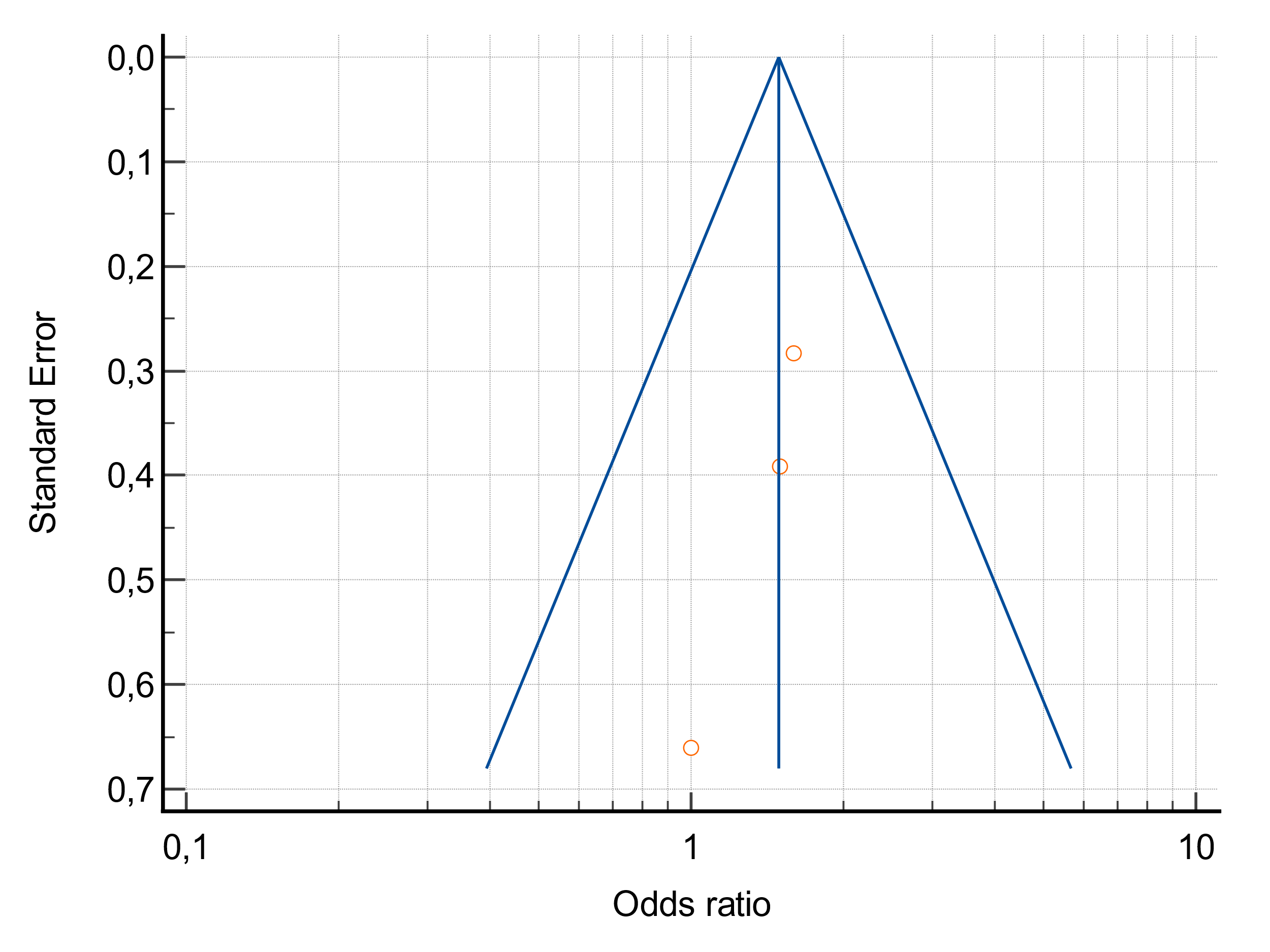


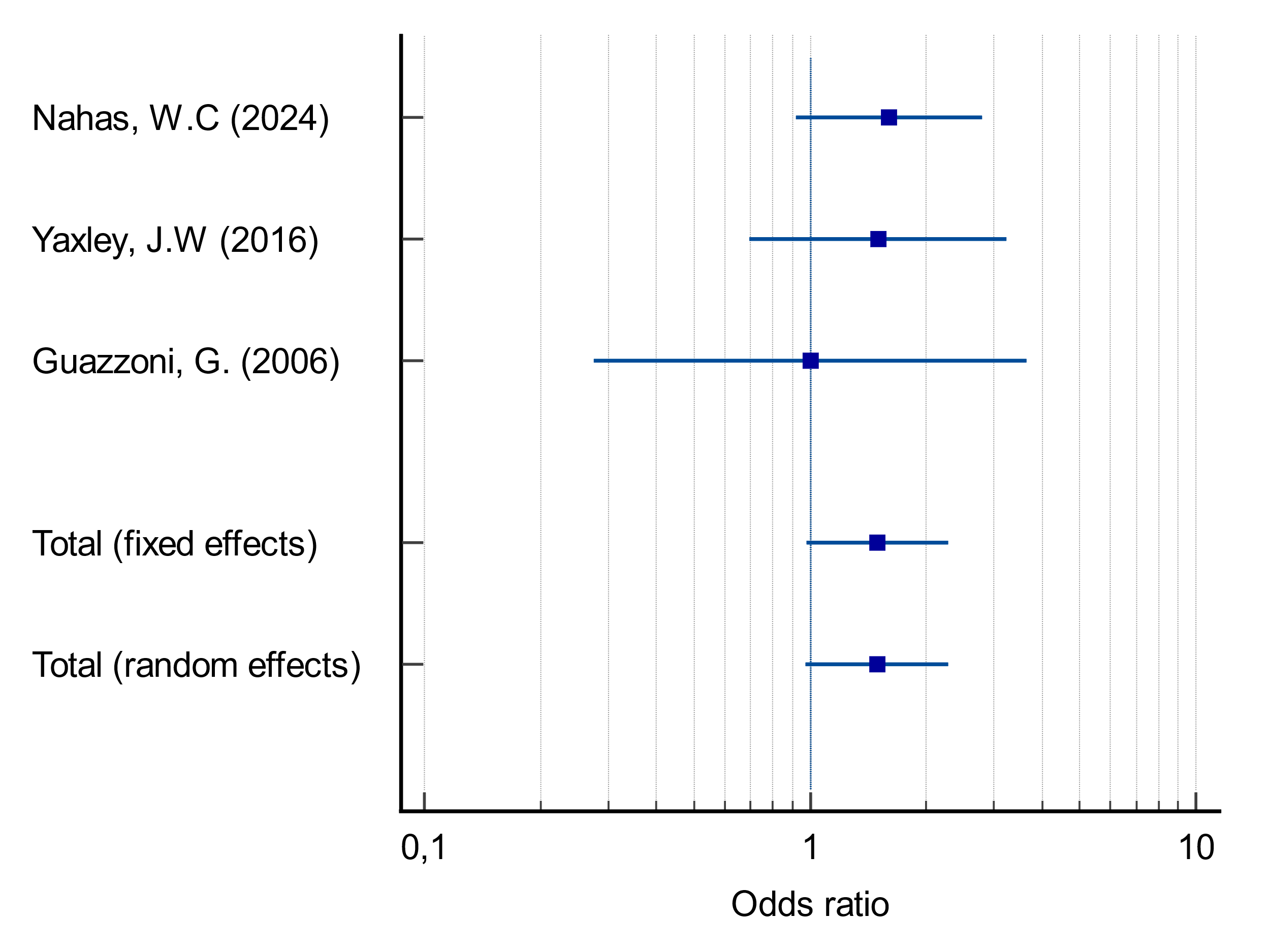


| Variable for studies | Artigo |
| --- | --- |
| 1. Intervention groups | |
| Variable for total number of cases | total_MIRP |
| Variable for number of positive cases | psmt3_MIRP |
| 2. Control groups | |
| Variable for total number of cases | total_ORP |
| Variable for number of positive cases | psmt3_ORP |

| Study | Intervention | Controls | Odds ratio | 95% CI | z | P | Weight (%) | |
| --- | --- | --- | --- | --- | --- | --- | --- | --- |
|  |  |  |  |  |  |  | Fixed | Random |
| Nahas, W.C (2024) | 40/171 | 25/156 | 1,600 | 0,918 to 2,788 |  |  | 58,56 | 58,56 |
| Yaxley, J.W (2016) | 18/157 | 12/151 | 1,500 | 0,696 to 3,231 |  |  | 30,67 | 30,67 |
| Guazzoni, G. (2006) | 5/60 | 5/60 | 1,000 | 0,274 to 3,650 |  |  | 10,77 | 10,77 |
| Total (fixed effects) | 63/388 | 42/367 | 1,492 | 0,976 to 2,280 | 1,849 | 0,064 | 100,00 | 100,00 |
| Total (random effects) | 63/388 | 42/367 | 1,491 | 0,975 to 2,281 | 1,843 | 0,065 | 100,00 | 100,00 |

**Test for heterogeneity**

| Q | 0,4279 |
| --- | --- |
| DF | 2 |
| Significance level | P = 0,8074 |
| I^2^ (inconsistency) | 0,00% |
| 95% CI for I^2^ | 0,00 to 84,32 |

**Publication bias**

| Egger's test | |
| --- | --- |
| Intercept | -1,1909 |
| 95% CI | -4,6674 to 2,2857 |
| Significance level | P = 0,1438 |
| Begg's test | |
| Kendall's Tau | -1,0000 |
| Significance level | P = 0,1172 |

**Urinary Continence at six months**
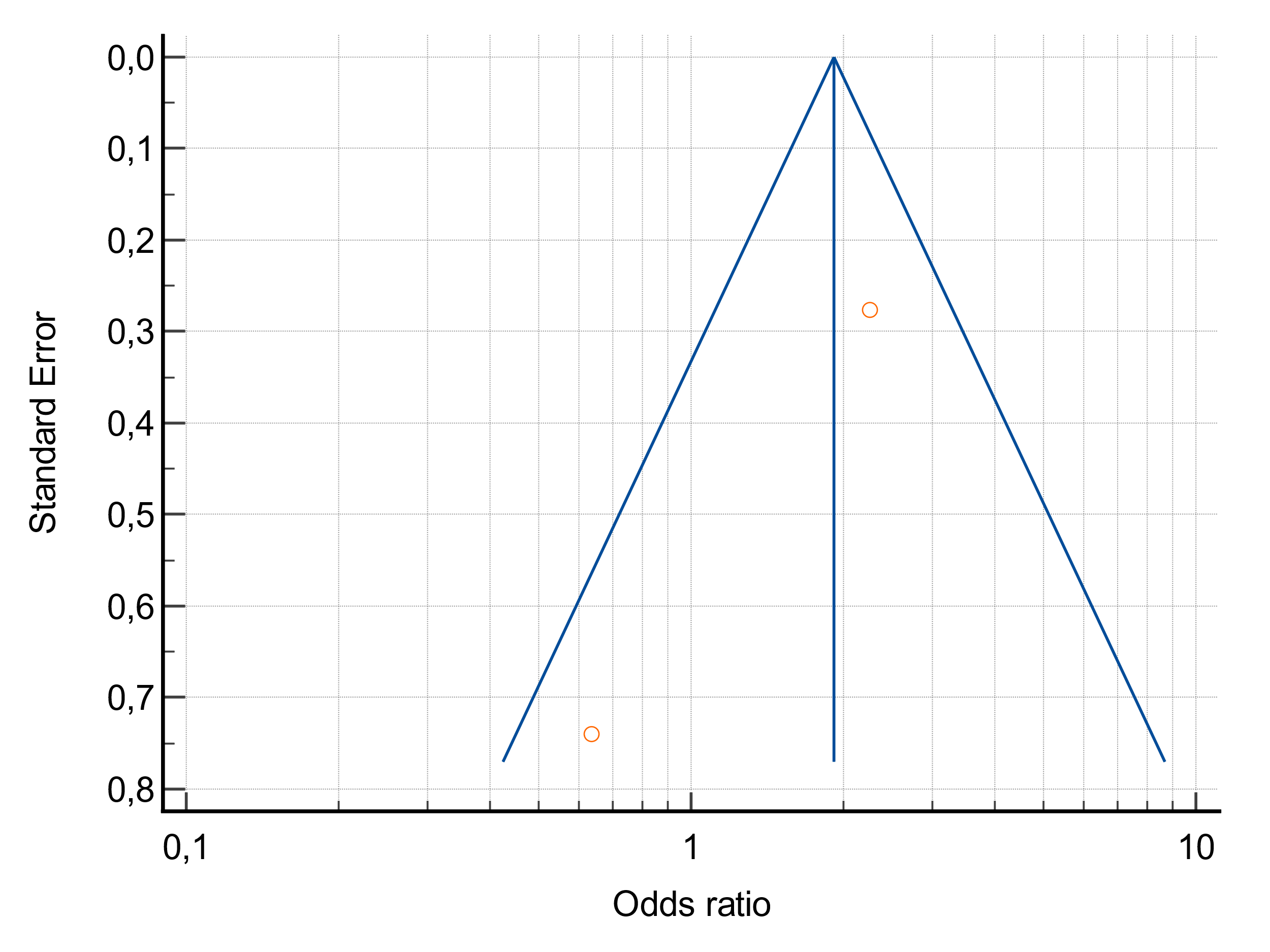

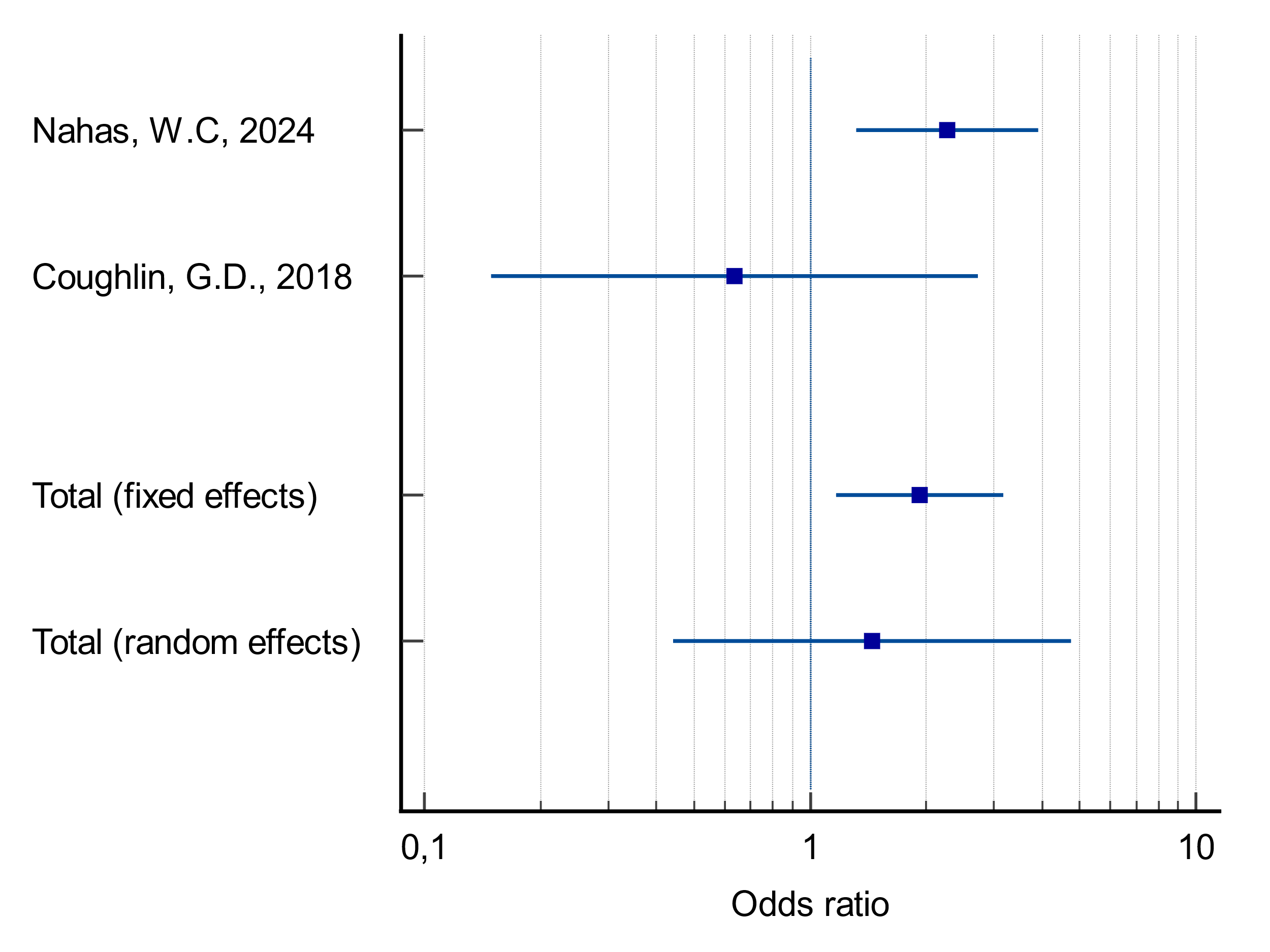


| Variable for studies | Study |
| --- | --- |
| 1. Intervention groups | |
| Variable for total number of cases | MIRP_total |
| Variable for number of positive cases | 6m_MIRP |
| 2. Control groups | |
| Variable for total number of cases | Open_total |
| Variable for number of positive cases | 6m_open |

| Study | Intervention | Controls | Odds ratio | 95% CI | z | P | Weight (%) | |
| --- | --- | --- | --- | --- | --- | --- | --- | --- |
|  |  |  |  |  |  |  | Fixed | Random |
| Nahas, W.C, 2024 | 145/171 | 111/156 | 2,261 | 1,314 to 3,889 |  |  | 87,74 | 64,67 |
| Coughlin, G.D., 2018 | 139/144 | 131/134 | 0,637 | 0,149 to 2,717 |  |  | 12,26 | 35,33 |
| Total (fixed effects) | 284/315 | 242/290 | 1,919 | 1,164 to 3,162 | 2,556 | 0,011 | 100,00 | 100,00 |
| Total (random effects) | 284/315 | 242/290 | 1,445 | 0,441 to 4,738 | 0,607 | 0,544 | 100,00 | 100,00 |

**Test for heterogeneity**

| Q | 2,5719 |
| --- | --- |
| DF | 1 |
| Significance level | P = 0,1088 |
| I^2^ (inconsistency) | 61,12% |
| 95% CI for I^2^ | 0,00 to 90,97 |

**Publication bias**

| Egger's test | |
| --- | --- |
| Intercept | -2,7335 |
| 95% CI |  |
| Significance level | P < 0,0001 |
| Begg's test | |
| Kendall's Tau | -1,0000 |
| Significance level | P = 0,3173 |

**Urinary Continence at 12 months**


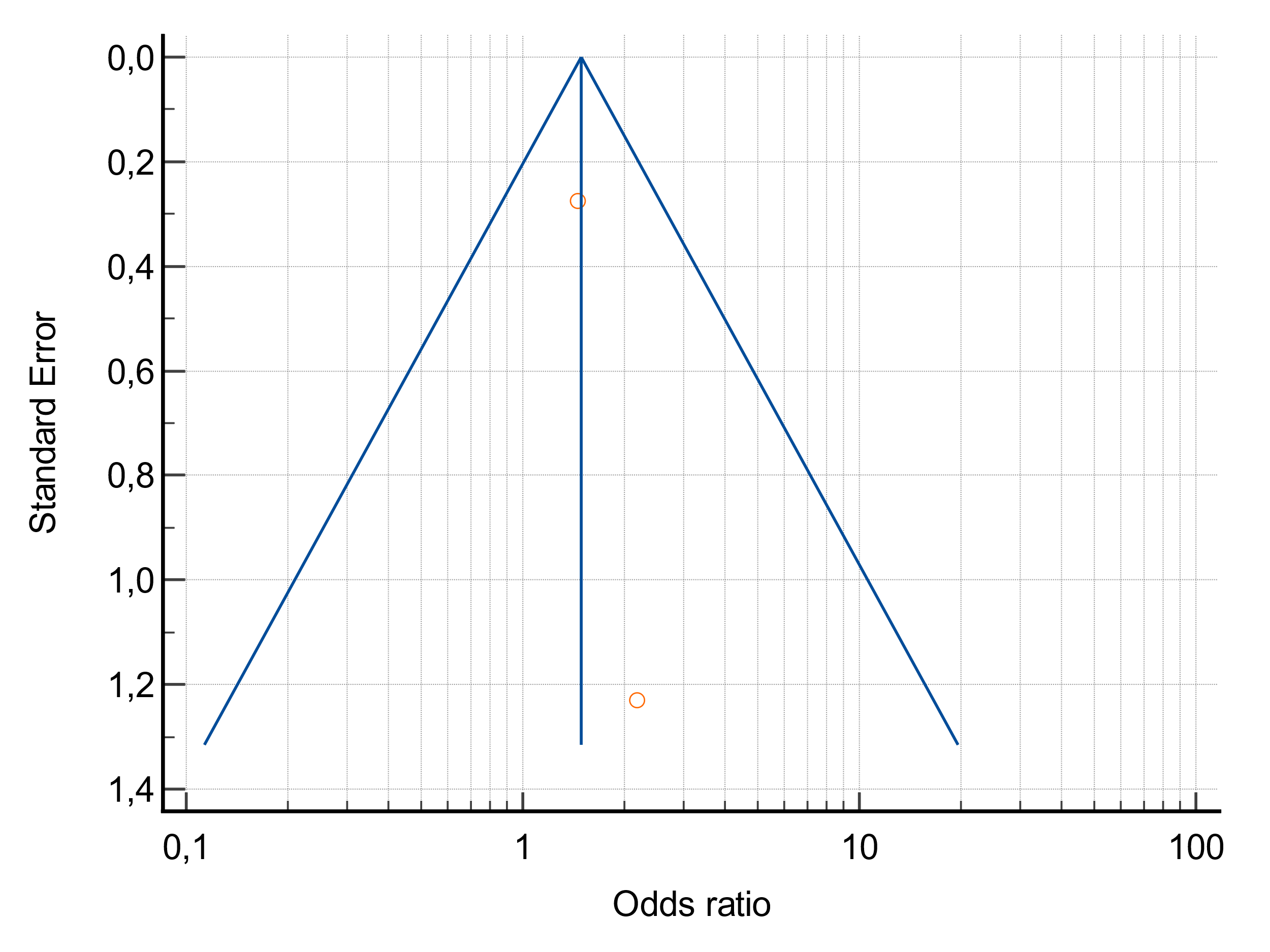

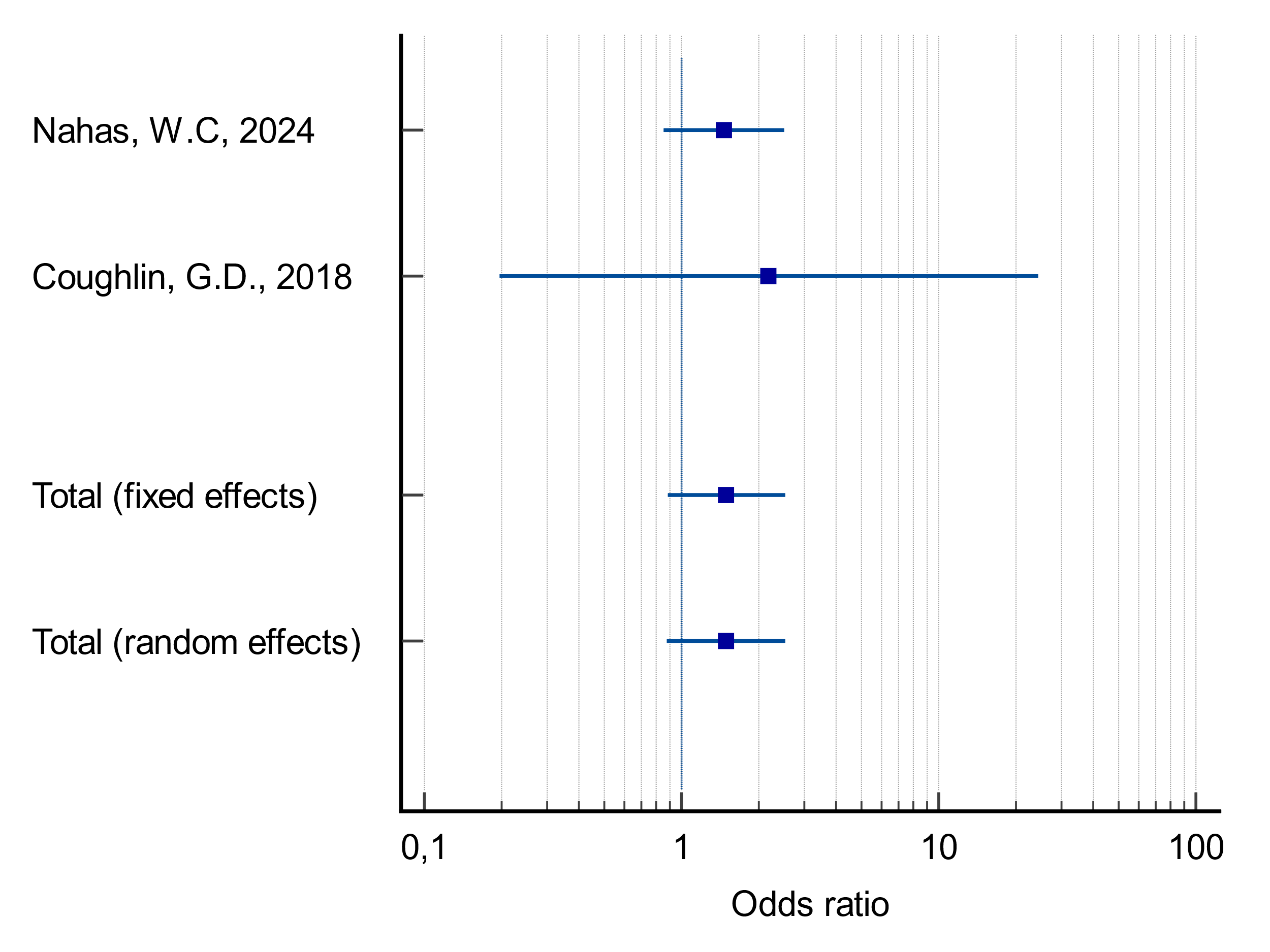


| Variable for studies | Study |
| --- | --- |
| 1. Intervention groups | |
| Variable for total number of cases | MIRP_total |
| Variable for number of positive cases | 12m_MIRP |
| 2. Control groups | |
| Variable for total number of cases | Open_total |
| Variable for number of positive cases | 12m_open |

| Study | Intervention | Controls | Odds ratio | 95% CI | z | P | Weight (%) | |
| --- | --- | --- | --- | --- | --- | --- | --- | --- |
|  |  |  |  |  |  |  | Fixed | Random |
| Nahas, W.C, 2024 | 141/171 | 119/156 | 1,461 | 0,852 to 2,507 |  |  | 95,23 | 95,23 |
| Coughlin, G.D., 2018 | 145/146 | 133/135 | 2,180 | 0,195 to 24,326 |  |  | 4,77 | 4,77 |
| Total (fixed effects) | 286/317 | 252/291 | 1,491 | 0,881 to 2,524 | 1,488 | 0,137 | 100,00 | 100,00 |
| Total (random effects) | 286/317 | 252/291 | 1,490 | 0,880 to 2,522 | 1,482 | 0,138 | 100,00 | 100,00 |

**Test for heterogeneity**

| Q | 0,1007 |
| --- | --- |
| DF | 1 |
| Significance level | P = 0,7510 |
| I^2^ (inconsistency) | 0,00% |
| 95% CI for I^2^ | 0,00 to 0,00 |

**Publication bias**

| Egger's test | |
| --- | --- |
| Intercept | 0,4189 |
| 95% CI |  |
| Significance level | P < 0,0001 |
| Begg's test | |
| Kendall's Tau | 1,0000 |
| Significance level | P = 0,3173 |

**Urinary Continence at longest follow-up**


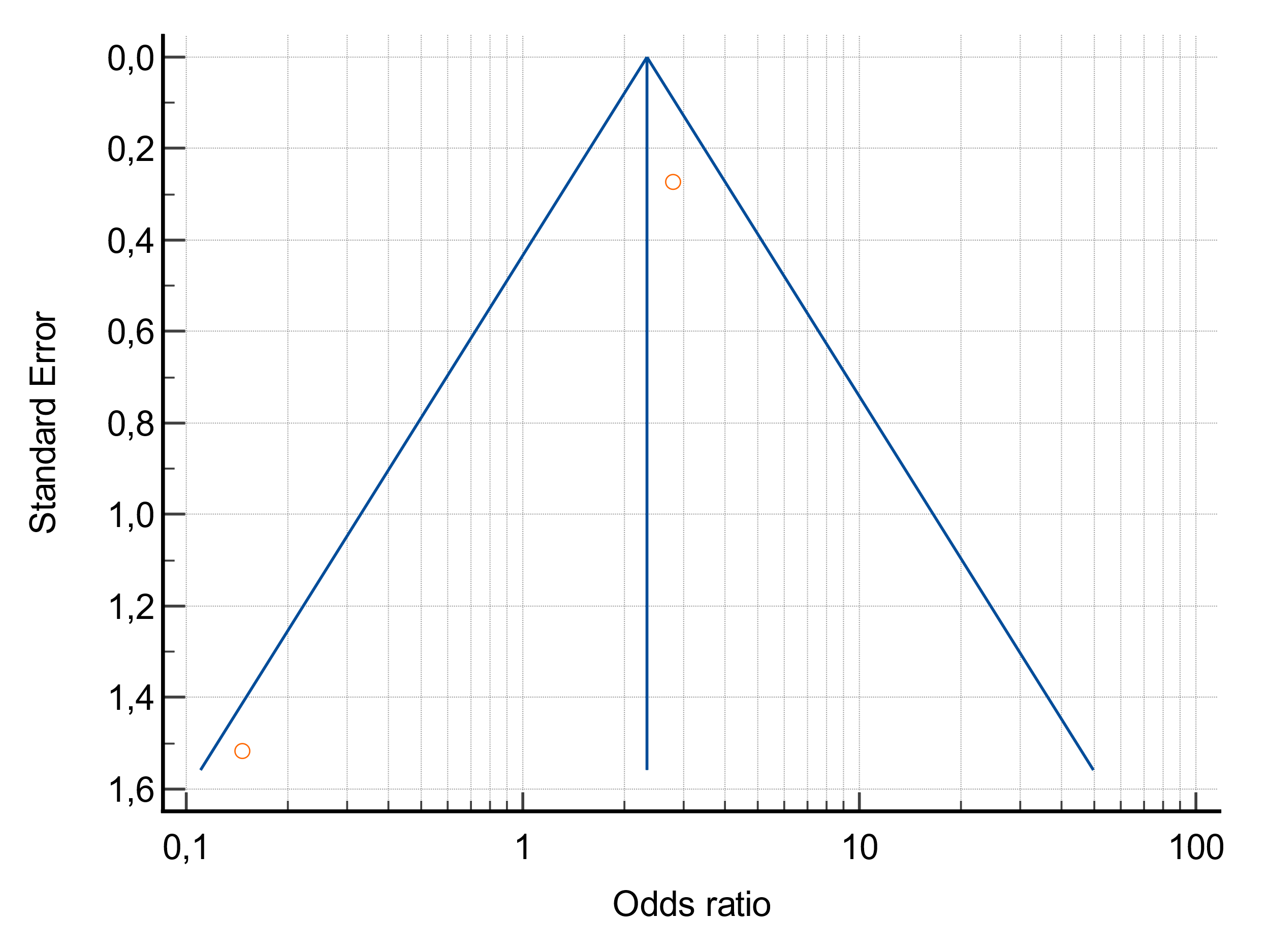


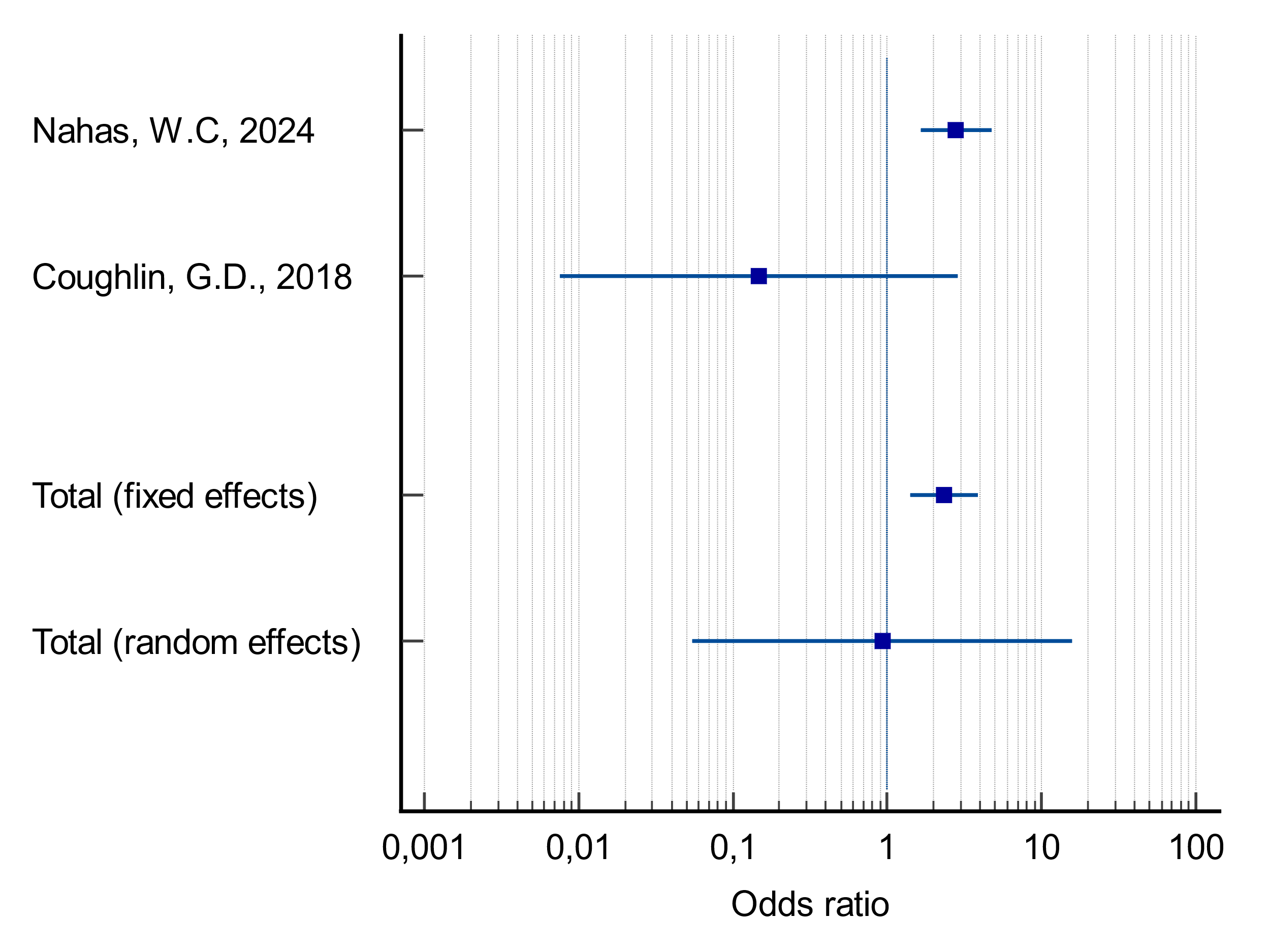


| Variable for studies | Study |
| --- | --- |
| 1. Intervention groups | |
| Variable for total number of cases | MIRP_total |
| Variable for number of positive cases | maximo_MIRP |
| 2. Control groups | |
| Variable for total number of cases | Open_total |
| Variable for number of positive cases | maximo_open |

| Study | Intervention | Controls | Odds ratio | 95% CI | z | P | Weight (%) | |
| --- | --- | --- | --- | --- | --- | --- | --- | --- |
|  |  |  |  |  |  |  | Fixed | Random |
| Nahas, W.C, 2024 | 145/171 | 104/156 | 2,788 | 1,635 to 4,756 |  |  | 96,88 | 62,53 |
| Coughlin, G.D., 2018 | 135/138 | 131/131 | 0,147 | 0,00753 to 2,878 |  |  | 3,12 | 37,47 |
| Total (fixed effects) | 280/309 | 235/287 | 2,338 | 1,412 to 3,873 | 3,300 | 0,001 | 100,00 | 100,00 |
| Total (random effects) | 280/309 | 235/287 | 0,926 | 0,0548 to 15,664 | -0,0532 | 0,958 | 100,00 | 100,00 |

**Test for heterogeneity**

| Q | 3,7415 |
| --- | --- |
| DF | 1 |
| Significance level | P = 0,0531 |
| I^2^ (inconsistency) | 73,27% |
| 95% CI for I^2^ | 0,00 to 93,98 |

**Publication bias**

| Egger's test | |
| --- | --- |
| Intercept | -2,3637 |
| 95% CI |  |
| Significance level | P < 0,0001 |
| Begg's test | |
| Kendall's Tau | -1,0000 |
| Significance level | P = 0,3173 |

**Erectile Function at six months**


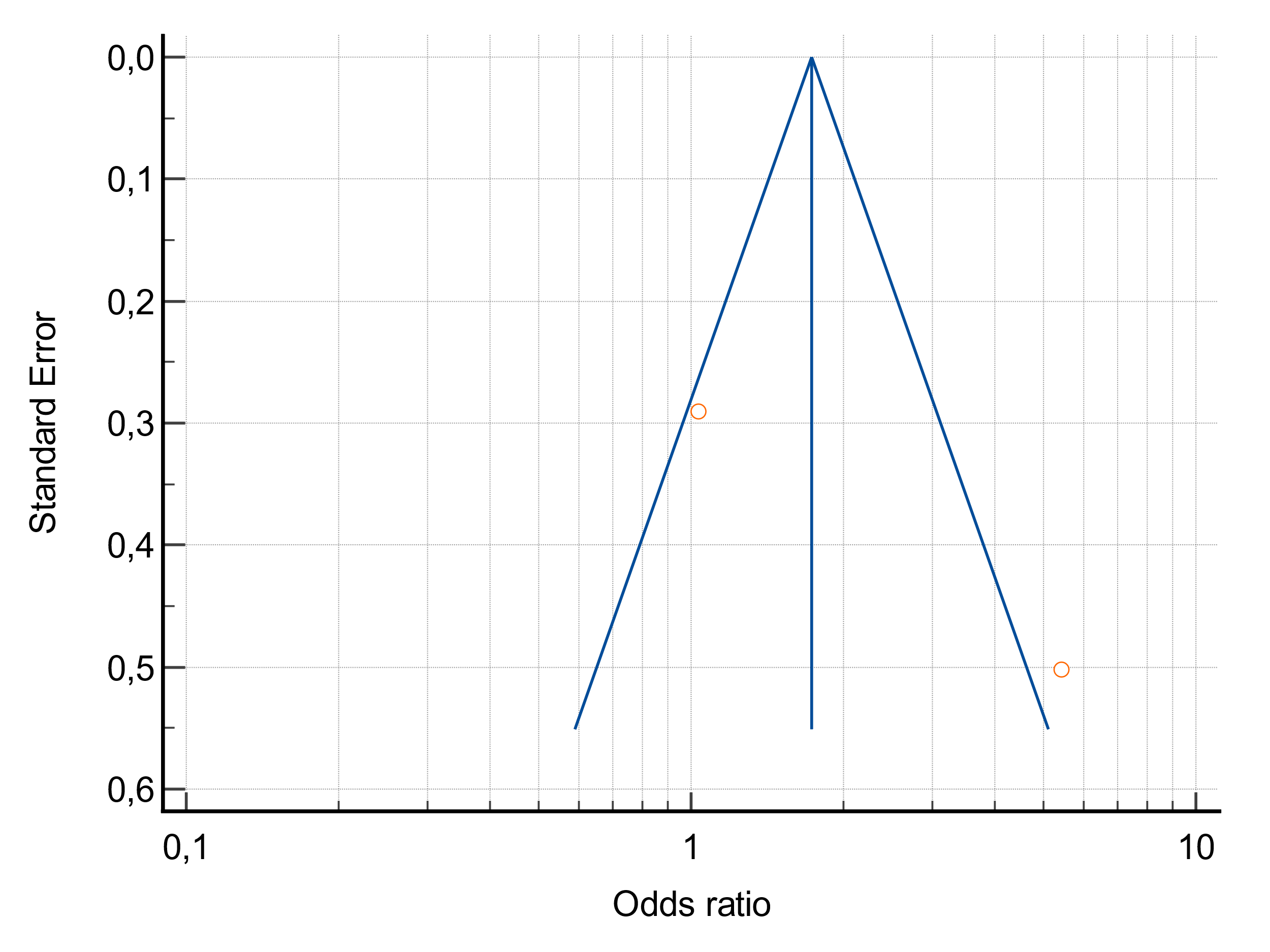


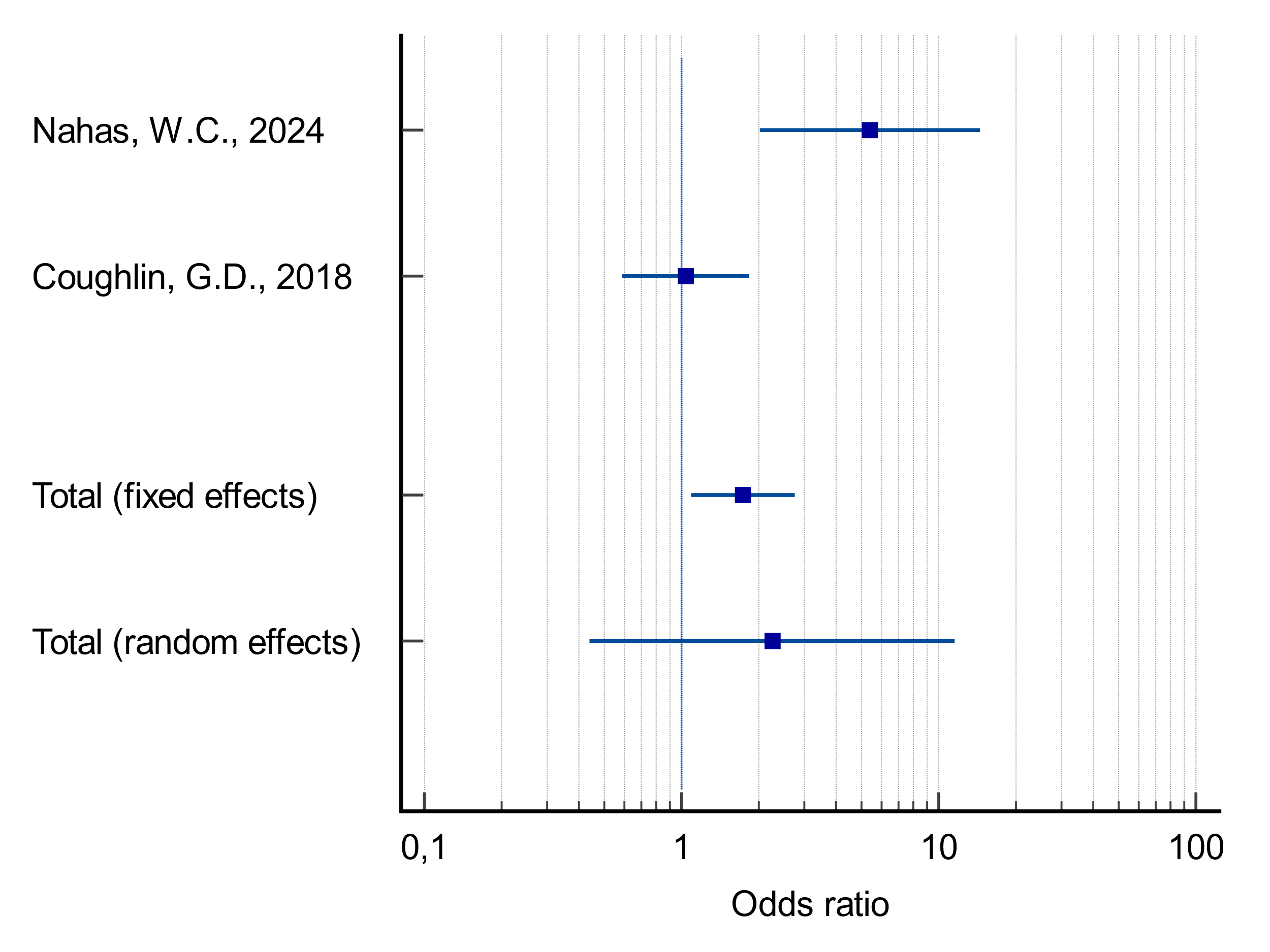


**Meta-analysis: odds ratio**

| Variable for studies | study |
| --- | --- |
| 1. Intervention groups | |
| Variable for total number of cases | MIRP_total MIRP+total |
| Variable for number of positive cases | funcaoeretil6m_MIRP |
| 2. Control groups | |
| Variable for total number of cases | open_total |
| Variable for number of positive cases | funcaoeretil6m_open |

| Study | Intervention | Controls | Odds ratio | 95% CI | z | P | Weight (%) | |
| --- | --- | --- | --- | --- | --- | --- | --- | --- |
|  |  |  |  |  |  |  | Fixed | Random |
| Nahas, W.C., 2024 | 26/171 | 5/156 | 5,415 | 2,025 to 14,484 |  |  | 25,04 | 47,00 |
| Coughlin, G.D., 2018 | 32/144 | 29/134 | 1,034 | 0,586 to 1,827 |  |  | 74,96 | 53,00 |
| Total (fixed effects) | 58/315 | 34/290 | 1,733 | 1,088 to 2,761 | 2,315 | 0,021 | 100,00 | 100,00 |
| Total (random effects) | 58/315 | 34/290 | 2,252 | 0,439 to 11,557 | 0,973 | 0,331 | 100,00 | 100,00 |

**Test for heterogeneity**

| Q | 8,3142 |
| --- | --- |
| DF | 1 |
| Significance level | P = 0,0039 |
| I^2^ (inconsistency) | 87,97% |
| 95% CI for I^2^ | 53,66 to 96,88 |

**Publication bias**

| Egger's test | |
| --- | --- |
| Intercept | 7,8143 |
| 95% CI |  |
| Significance level | P < 0,0001 |
| Begg's test | |
| Kendall's Tau | 1,0000 |
| Significance level | P = 0,3173 |

**Erectile Function at 12 months**


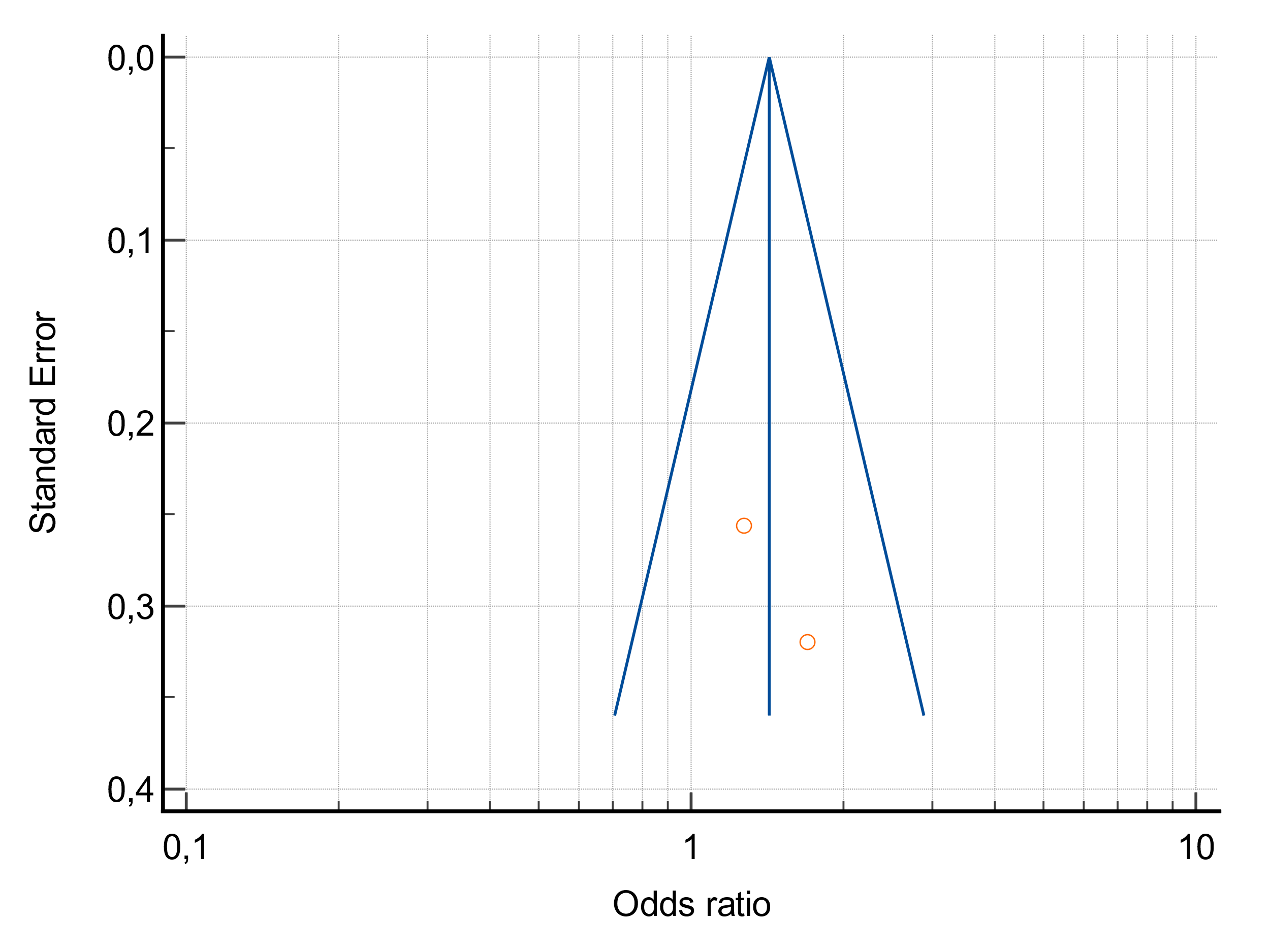


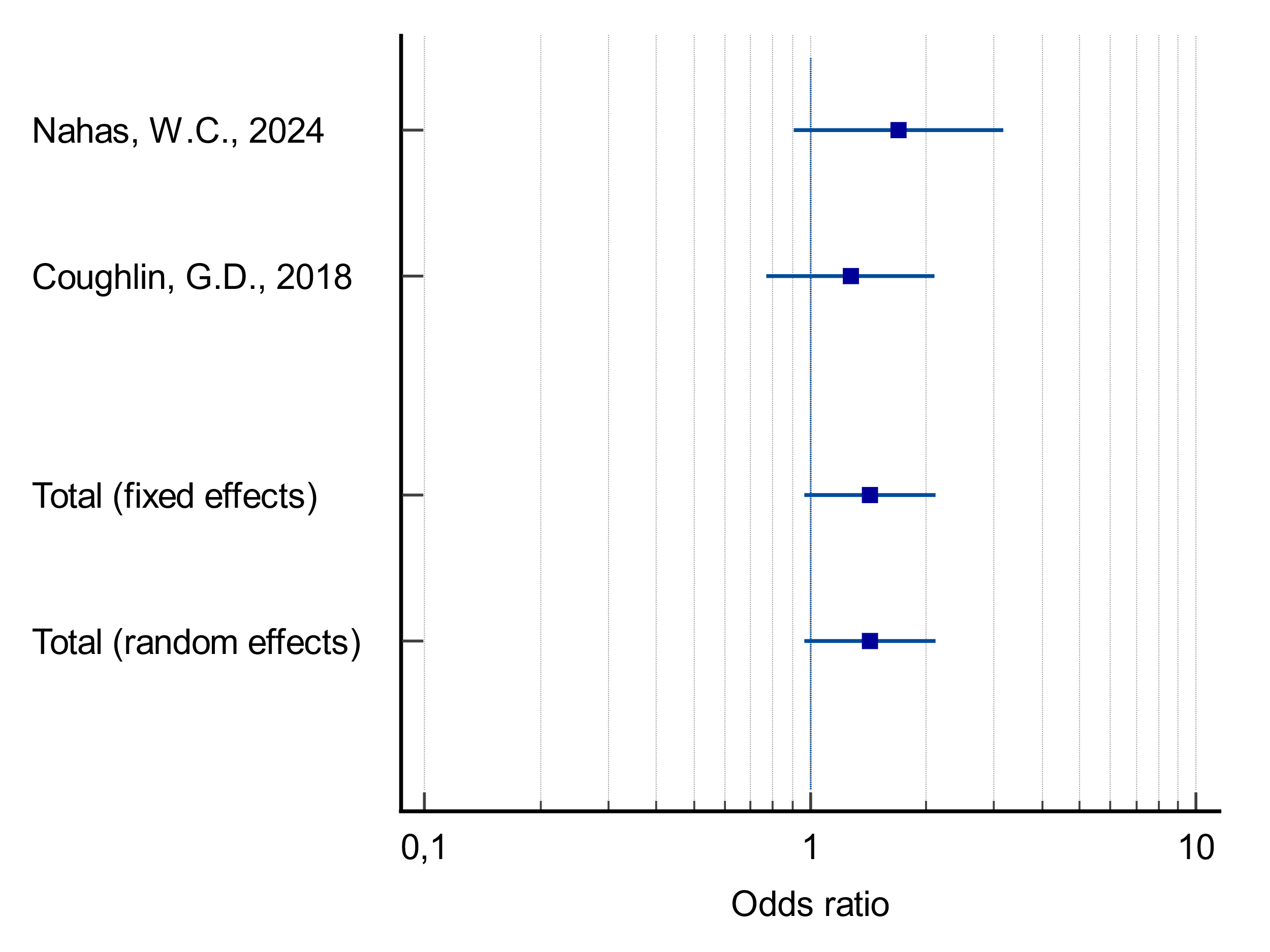


**Meta-analysis: odds ratio**

| Variable for studies | study |
| --- | --- |
| 1. Intervention groups | |
| Variable for total number of cases | MIRP_total MIRP+total |
| Variable for number of positive cases | funcaoeretil12m_MIRP |
| 2. Control groups | |
| Variable for total number of cases | open_total |
| Variable for number of positive cases | funcaoeretil12m_open |

| Study | Intervention | Controls | Odds ratio | 95% CI | z | P | Weight (%) | |
| --- | --- | --- | --- | --- | --- | --- | --- | --- |
|  |  |  |  |  |  |  | Fixed | Random |
| Nahas, W.C., 2024 | 31/171 | 18/156 | 1,698 | 0,907 to 3,177 |  |  | 39,12 | 39,12 |
| Coughlin, G.D., 2018 | 51/146 | 40/135 | 1,275 | 0,772 to 2,107 |  |  | 60,88 | 60,88 |
| Total (fixed effects) | 82/317 | 58/291 | 1,428 | 0,966 to 2,112 | 1,788 | 0,074 | 100,00 | 100,00 |
| Total (random effects) | 82/317 | 58/291 | 1,426 | 0,964 to 2,110 | 1,775 | 0,076 | 100,00 | 100,00 |

**Test for heterogeneity**

| Q | 0,4883 |
| --- | --- |
| DF | 1 |
| Significance level | P = 0,4847 |
| I^2^ (inconsistency) | 0,00% |
| 95% CI for I^2^ | 0,00 to 0,00 |

**Publication bias**

| Egger's test | |
| --- | --- |
| Intercept | 4,5120 |
| 95% CI |  |
| Significance level | P < 0,0001 |
| Begg's test | |
| Kendall's Tau | 1,0000 |
| Significance level | P = 0,3173 |
